# Supplementary material for: Possible factors determining global-scale patterns of crop yield sensitivity to drought
Source: PLoS One. 2023 Feb 2;18(2):e0281287. doi: 10.1371/journal.pone.0281287 (PMC9894396; doi:10.1371/journal.pone.0281287)
Supplement: S1 Table — All datasets are freely available via websites. (PDF) [file pone.0281287.s001.pdf]

S1 Table. List of dataset sources. All datasets are freely available via websites.

Class	Variable	Website source	Format
Climate	Mean annual precipitation	https://crudata.uea.ac.uk/cru/data/hrg/cru_ts_4.06/cruts.2205201912.v4.06/	netCDF
	Mean annual PET		
	Mean temperature		
Terrain	Elevation	https://www.fao.org/fileadmin/user_upload/soils/HWSD%20Viewer/GloElev_30as.rar	ASCII
	Slope		
Soil	Topsoil saturated hydraulic conductivity	https://doi.pangaea.de/10.1594/PANGAEA.870605	netCDF
	Topsoil clay amount	https://daac.ornl.gov/cgi-bin/dsviewer.pl?ds_id=1247	netCDF
	Topsoil organic carbon		
	Topsoil acidity		
	Water-holding capacity	https://daac.ornl.gov/cgi-bin/dsviewer.pl?ds_id=548	ASCII
Irrigation	Area equipped for irrigation (AEI)	https://www.fao.org/aquastat/en/geospatial-information/global-maps-irrigated-areas/latest-version	ASCII
	Area actually irrigated (AAI)		
	Area irrigated with groundwater (AEIGW)		
	Area irrigated with water from non-conventional sources (AEINC)		
Production	Growing season length	https://sage.nelson.wisc.edu/data-and-models/datasets/crop-calendar-dataset/netcdf-0-5-degree/	netCDF
	Harvested area	https://hessenbox-a10.rz.uni-frankfurt.de/getlink/fi72QzqeLV6gHKy49zUSnP5X/harvested_area_grids	ASCII
Fertilizer	Nitrogen rate application	http://www.earthstat.org/nutrient-application-major-crops/	GeoTIFF
	Phosphorus rate application		
	Potassium rate application		
Socioeconomic	GDP	https://datadryad.org/stash/dataset/doi:10.5061/dryad.dk1j0	netCDF
	GDP per capita		
	Population	https://sedac.ciesin.columbia.edu/data/set/gpw-v4-population-density-rev11	netCDF
