# Supplementary material for: Possible factors determining global-scale patterns of crop yield sensitivity to drought
Source: PLoS One. 2023 Feb 2;18(2):e0281287. doi: 10.1371/journal.pone.0281287 (PMC9894396; doi:10.1371/journal.pone.0281287)
Supplement: S2 Table — The support vector machine model is built using ‴svmRadial‴ based on the "caret" R library with the parameter sigma value (σ) of 0.1 and cost (C) of 1. Extreme gradient boosting is developed using "xgbTree" method in "caret" using its default fitting parameters (e.g., number of rounds of boosting = 500, maximum depth of a tree = 6, learning rate = 0.3). The values of the performance skills are based on the mean value from 10-fold cross-validation, except in the case of random Forest, which is solely based on OOB (out-of-bag) validation in this study. The unit percentage for RMSE denotes crop yield anomaly per drought magnitude (β). (PDF) [file pone.0281287.s002.pdf]

S4 Table. Output performance (R^2 and RMSE) of each machine learning algorithm trained in this study. The support vector machine model is built using '"svmRadial"' based on the "caret" R package with the parameter sigma value (σ) of 0.1 and cost (C) of 1. Extreme gradient boosting is developed using "xgbTree" method in "caret" using its default fitting parameters (e.g., number of rounds of boosting = 500, maximum depth of a tree = 6, learning rate = 0.3). The values of the performance skills are based on the mean value from 10-fold cross-validation, except in the case of random Forest, which is solely based on OOB (out-of-bag) validation in this study.

Machine learning algorithm	Maize		Rice		Soybean		Wheat	
	R^2	RMSE	R^2	RMSE	R^2	RMSE	R^2	RMSE
Random Forest	0.44	8.61%	0.36	5.89%	0.66	4.63%	0.37	10.01%
Support Vector Regression	0.44	8.60%	0.33	6.04%	0.61	5.00%	0.39	9.83%
Extreme Gradient Boosting	0.42	8.85%	0.31	6.27%	0.62	4.96%	0.34	10.53%
Mean	0.43	8.69%	0.33	6.07%	0.63	4.86%	0.37	10.12%
