# Supplementary figures and images for: Possible factors determining global-scale patterns of crop yield sensitivity to drought
Source: PLoS One. 2023 Feb 2;18(2):e0281287. doi: 10.1371/journal.pone.0281287 (PMC9894396; doi:10.1371/journal.pone.0281287)

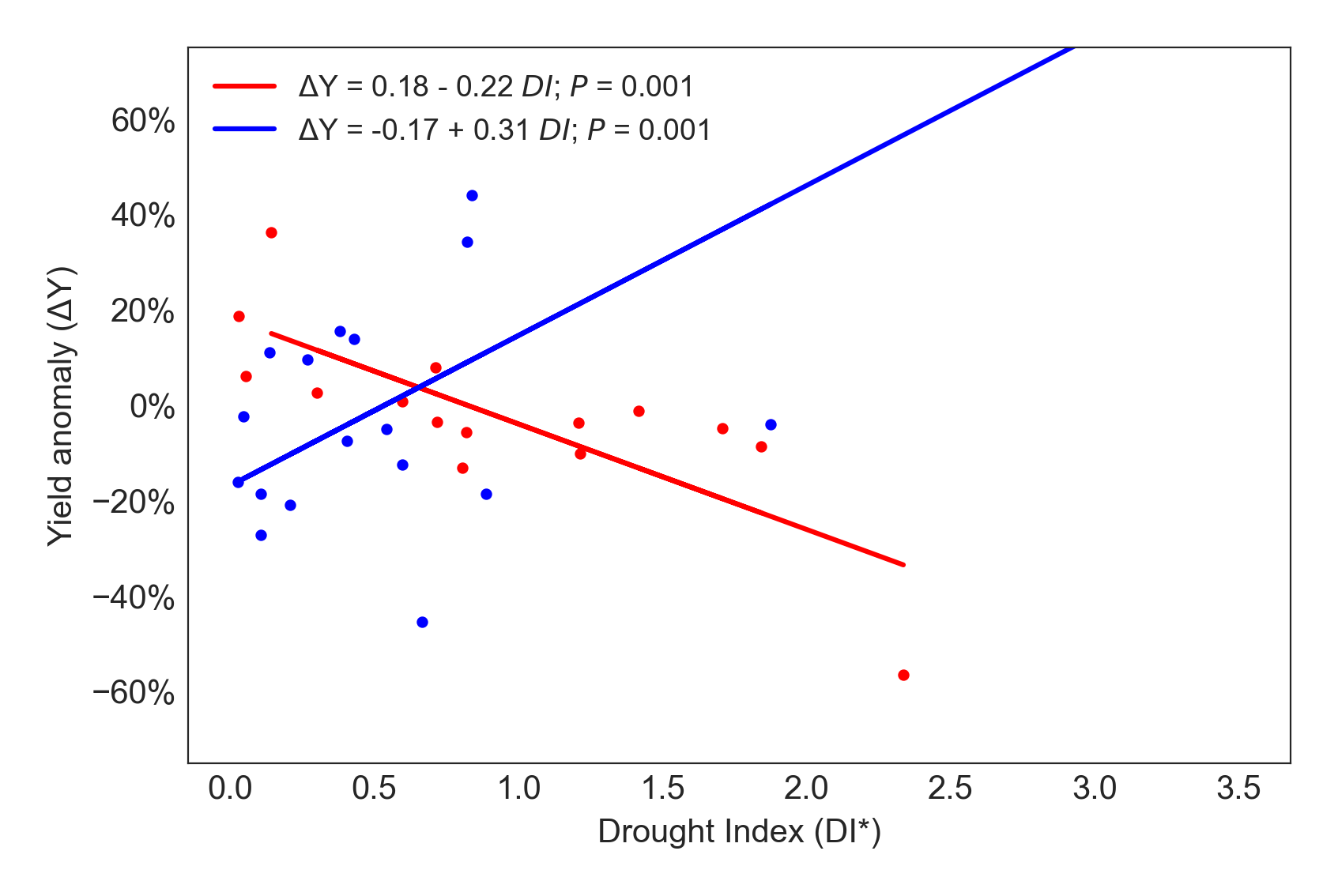

Supplement: S1 Fig — Crop drought sensitivity (β) is obtained based on the slope coefficient. (TIF) [file pone.0281287.s003.tif]

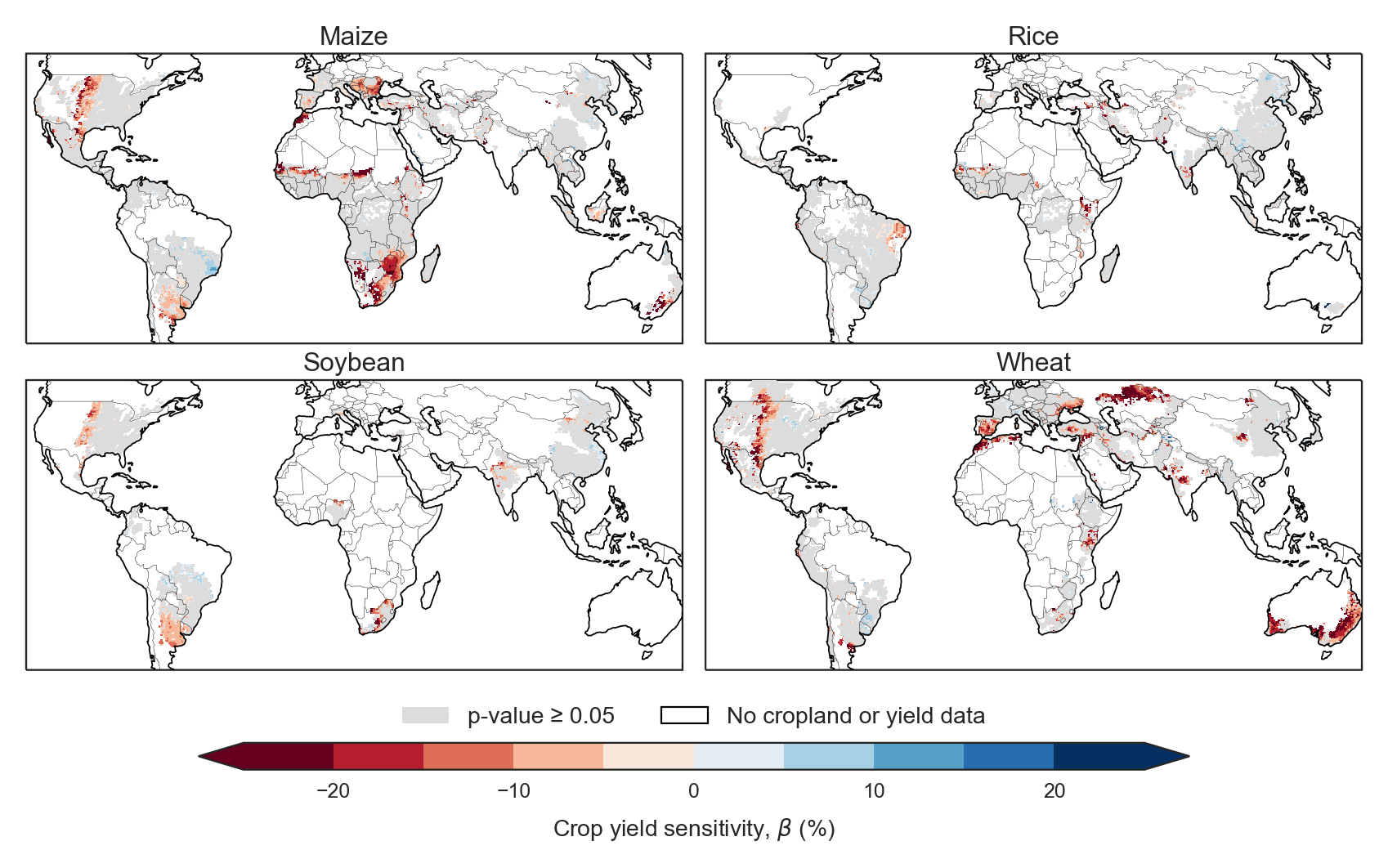

Supplement: S2 Fig — Sensitivity is calculated by the linear slope coefficient (β) with crop yield anomaly (%) per drought magnitude. White grid cells show no cropland or crop yield data, and grey shows the non-significant grid cells (P ≥ 0.05). (TIF) [file pone.0281287.s004.tif]

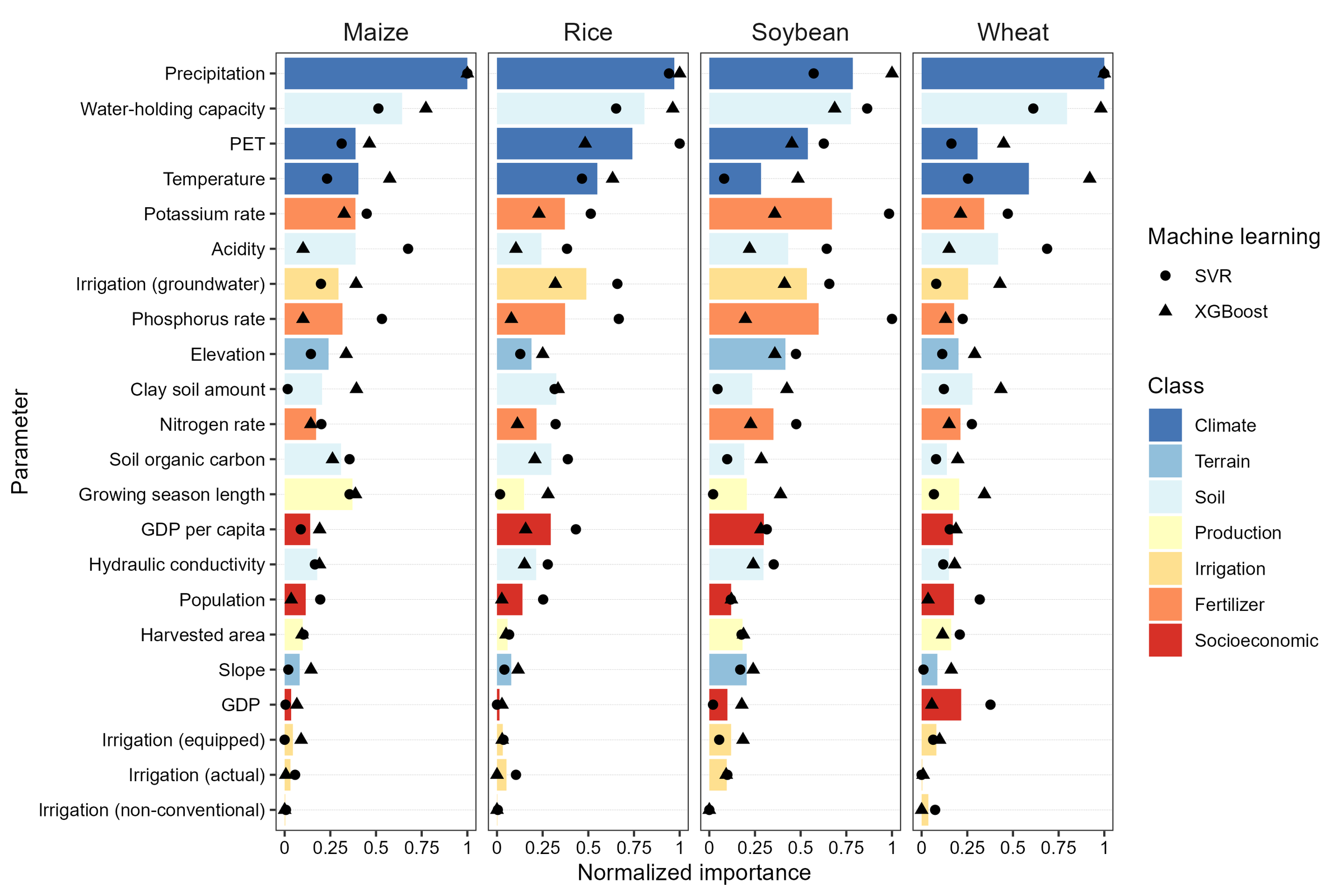

Supplement: S3 Fig — The importance values are normalized to the 0–1 range computed separately across different models. The colored bars represent the average relative importance across different algorithms. Label order of the y-axis indicates the order from the most important variables to the least based on the overall average across crops and models. (TIF) [file pone.0281287.s005.tif]

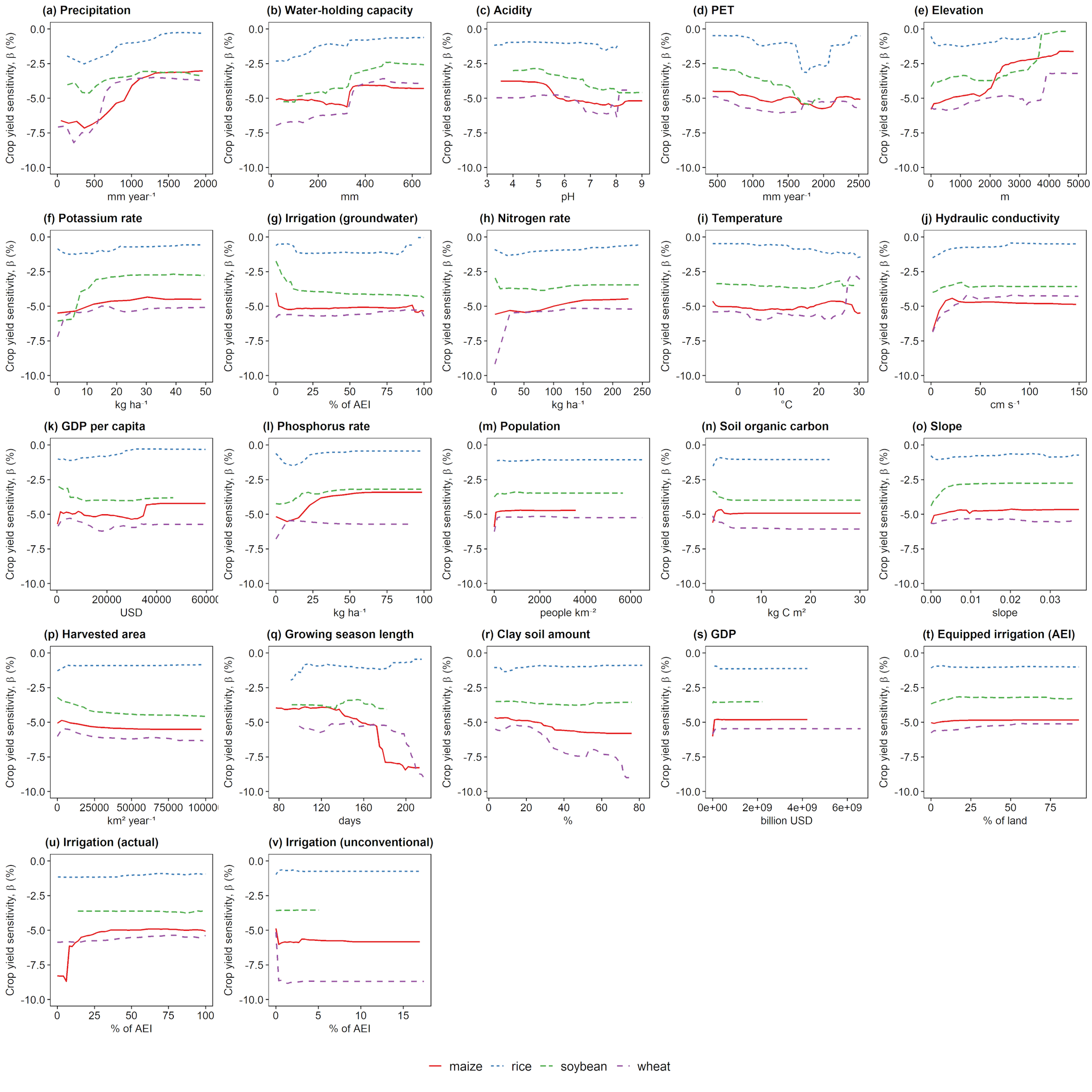

Supplement: S4 Fig — The order shows high to lower average relative importance across crops based on this specific model (not overall). The lower negative y-axis indicates higher yield loss due to drought. (TIF) [file pone.0281287.s006.tif]

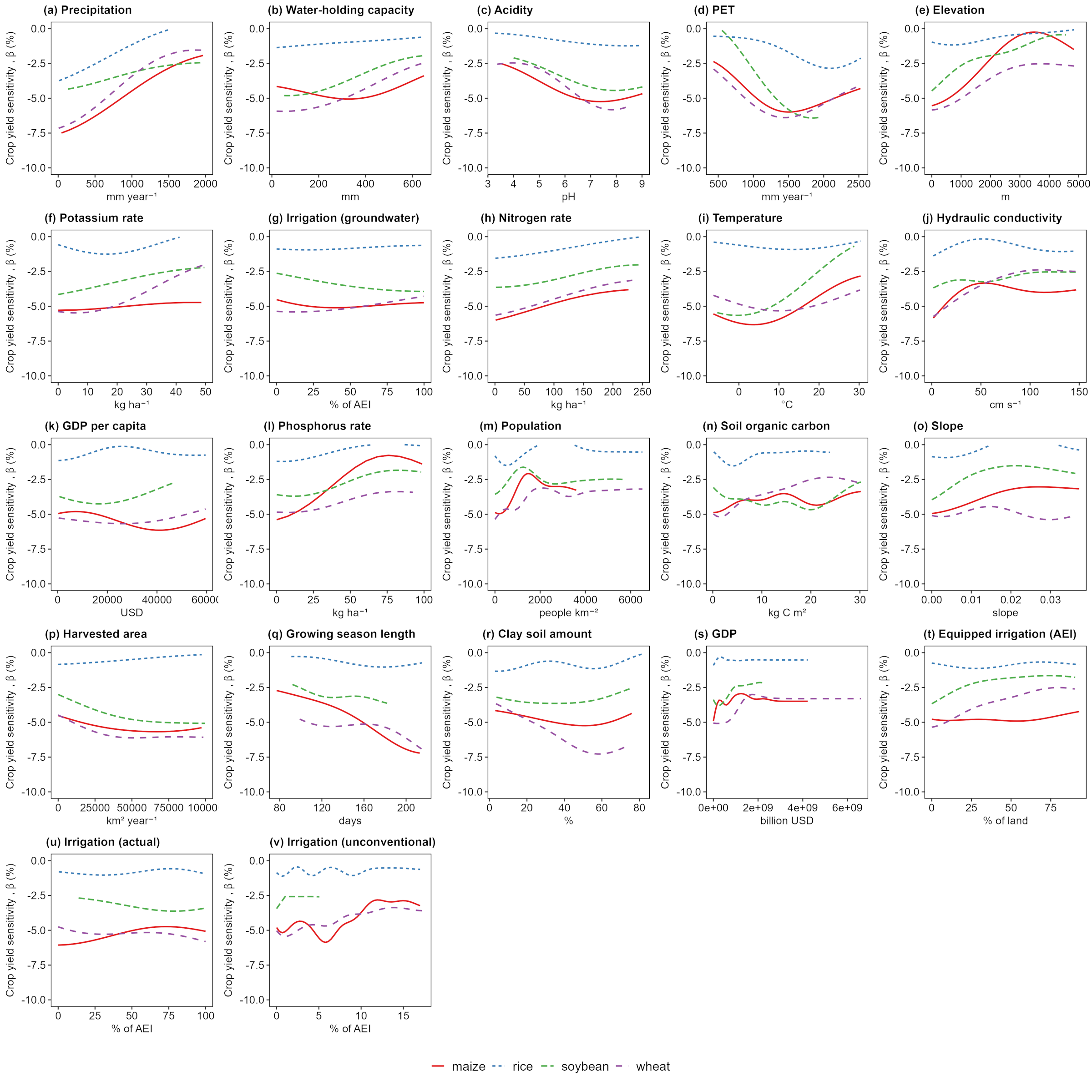

Supplement: S5 Fig — (TIF) [file pone.0281287.s007.tif]

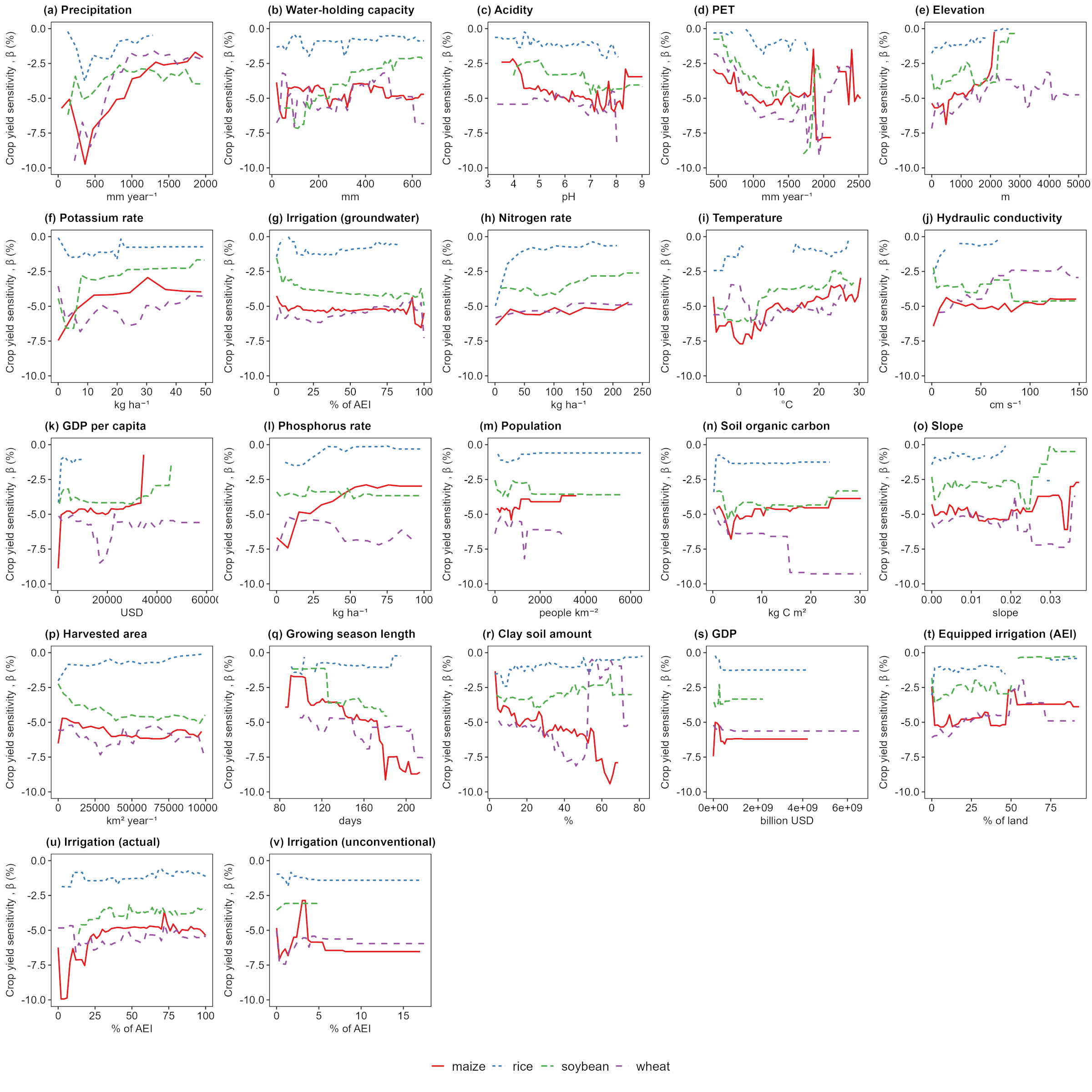

Supplement: S6 Fig — (TIF) [file pone.0281287.s008.tif]
