# Supplementary material for: Possible factors determining global-scale patterns of crop yield sensitivity to drought
Source: PLoS One. 2023 Feb 2;18(2):e0281287. doi: 10.1371/journal.pone.0281287 (PMC9894396; doi:10.1371/journal.pone.0281287)
Supplement: Attachment — Submitted filename: Comments and review for manuscript PONE-D-22-24361.pdf [file pone.0281287.s010.pdf]

Comments and review for manuscript **Number PONE-D-22-24361**

The submitted manuscript investigated possible factors determining spatial pattern of drought impact on major crop yield sensitivity on a global scale

The overall framework of the study was well planned with enough information about the subject matter. Here are some specific comments with the hope that will help to clarify some things and improve the manuscript overall.

1. The authors did not express their aim well enough in the last paragraph of the introduction section and should be written more clearly about how the results of the study will contribute to the literature.
2. Should be added to more literature related to this study in the introduction section.
3. Line 182 – line 183 and Line 196 – line 201 Authors should revise the results section. There seems to be discussing rather than pointing out the result of the study.
4. Line 335 – line 347 Authors should rewrite and present them as results.
5. Authors should be more detailed about the data analysis session. The tool used for the analysis should be stated clearly with references.
6. Authors should make the results session come out clear and on point.
7. Authors should improve the discussion section and avoid repetition of results in the discussion section. More literature should be included to make it solid.
8. Conclusion section should be shortened and concrete.
9. The references section should be double-checked for the journal formatting style and rules.
