# Supplementary material for: Possible factors determining global-scale patterns of crop yield sensitivity to drought
Source: PLoS One. 2023 Feb 2;18(2):e0281287. doi: 10.1371/journal.pone.0281287 (PMC9894396; doi:10.1371/journal.pone.0281287)
Supplement: Attachment — Submitted filename: PONE-D-22-24361_response-reviewers.pdf [file pone.0281287.s011.pdf]

Manuscript PONE-D-22-24361

Dear respected editor and reviewers,

Thank you for allowing us to improve our manuscript for publication in PLOS ONE. We appreciate the time and effort that the editor and reviewers dedicated to providing feedback on our manuscript. This document responds to each comment and suggestion raised by the academic editor and reviewer(s).

Together with this document, we have uploaded the revised manuscript with and without track changes.

A point-by-point response to the editor's comments
(copy of the ones written in the cover letter)**:**

1. Regarding the formatting style, we have revised our manuscript to meet PLOS ONE's style requirements provided (i.e., format of the main body and author and affiliations). Check our current revised manuscript.

2. Regarding data availability, we confirm that our data has no restrictions. We have uploaded the data set necessary to replicate our study findings to a public repository with the following DOI:

   10.6084/m9.figshare.21298785

   We have also added the list of external dataset sources in S1 Table in the revised manuscript. The order or Supplementary files are adjusted accordingly due to this addition.

3. We acknowledge that Figures 1 and S2 (S3 after revised) in our article contain third-party base map images. However, we ensure they are all freely available under the public domain.

   We have added the proper attribution in the Figure caption on our revised manuscript as follows.

   "The map was made with Cartopy library in Python (version 0.17) and the Natural Earth free vector and raster map data (naturalearthdata.com), which are freely available through the public domain..."

A point-by-point response to the reviewer's comments:

The line numbers mentioned in this document refer to the line number in the manuscript with track changes.

Reviewer #1:

We have accepted all the minor corrections from Reviewer #1 indicated in the word document. Thank you for your careful check.
Below are point-by-point responses to each comment..

1. Should be decades, as drought has been accompanying climate change for over 50 years

   Thank you for your corrections. We have revised the sentence accordingly as reviewer suggested (see **Line 18** in the Revised Manuscript with Track Changes).

2. What is a region, is that a specific region or you meant, regionally and globally?

   Yes, it should be "regionally". We have revised it in the current manuscript. (**Line 19**)

3. No need to say "previous studies" in the abstract, when the statement is general, such as the one here, especially you are citing none

   Thank you. We have corrected the abstract, changing it with the sentence as follows.

   Lines 19-21
   "It was estimated that crop yield variability in around one-third to three-fourths of global harvested areas is explained significantly by drought…"

4. Unclear of the value that this statement adds to the abstract, rephrase

   We finally removed the sentence to avoid further confusion (see **Lines 28-31**).

5. See https://www.nature.com/articles/s41598-022-09611-0
   Better stay clear of statements such as the first, the best, the top, etc., when describing your work

   Thank you. We acknowledge this. We have updated this part in the abstract as follows.

   Lines 28-32
   "This study quantitatively assesses the possible effect of various determinants which might control crop vulnerability to drought."

6. Drought stress?

   We have revised the sentence as follows.

   Lines 32-33
   "This understanding may provide insights for further studies addressing better crop-drought vulnerability measures under future drought stress."

7. What range is the "current period"

We have changed the word with “during the last decades” according to the study referenced and similar to that revised in the abstract (see **Line 40**).

8. Range?

We have also changed “in the current period” to “from 1983 to 2009” according to the study referenced (see **Line 43**).

9. You either state this as "drought" or "climate-related stressors"

Thank you for your correction, we changed it to “climate-related stressors” as reviewer suggested (see **Line 21** and **Line 45**).

10. Crop or farm management? Or other management? How about crop inputs, revenue, and other economic variables?

We updated “management” to “crop management” and “fertilizer input” to “agricultural input”. We also have added farmer income in addition to GDP per capita for extended examples of economic variables as suggested (see **Lines 61-62**).

11. What makes these systems more resilient?

We have updated the sentence as follows.
Lines 66-68
Current reports also revealed that more resilient agricultural systems, due to social, institutional and agroecological factors [23], could adapt to stress and recover better than those less resilient, even with a comparable or higher climate extreme exposure [20,22].

12. Mention the gap, reader at this stage needs to have a full sentence

We now explicitly mention the gap in the revised paragraph. See the sentence as follows.

Lines 82-83
“This study addresses the above gaps of lacking quantitative evidence of the effect of multiple factors in determining global crop-drought vulnerability based on finer gridded historical data...”

13. You must acknowledge the type of each and sources of the data

We have added the list of data sources and their type in Supplementary table S1. We update the sentence in **Line 96** and indicate the S1 Table information in the caption of Table 1 (see **Lines 153-154**).

14. Spell out

We have spelled out the abbreviations. See **Lines 104-105** in the revised manuscript.

15. Crop type? (**Line 144**)

Here, what we meant is crop production factors (i.e., growing season length harvested area) as mentioned in Table 1.

16. Still does not explain the lower rice response to drought in the model

We have improved the explanation in the revised manuscript (see as follows).

Lines 230-232
"In rice, where the models less explain the crop-drought sensitivity variation, there are likely other more complex effects of factors contributing to rice vulnerability to drought [1,7]."

17. Elaborate under the table on what meant by population (Table 2)

It refers to population density (people/km2). We further clarified it in Table 1 and updated the term "population density" throughout the manuscript.

18. In the previous paragraph, temp had a slight impact on response variables, how do you explain this contradiction

Thank you for your point. Yes, we agree. The general explanation stated does not correspond with the other study result provided in the previous paragraph. We have carefully updated it, preventing a possible contradiction while reading the paragraph (see **Lines 272-275**).

19. Soil salinity usually accompany drought, especially in irrigated lands, needs to be explored as well (**Line 278**)

We acknowledge that soil salinity may affect crop sensitivity to drought and needs to be explored. However, in this current study, we have not included the soil salinity parameter explicitly as one of the factors. One of the reasons is that there is quite a limited existing observation dataset on a global scale, though it can be estimated by several approaches such as in Ivushkin et al. (2019). One of the available datasets is the WoSIS Soil Profile Database (Ribeiro et al., 2015; Ivushkin et al., 2019). However, the dataset has undistributed samples which possibly adds more uncertainty to this study. Moreover, soil salinity is a quite dynamic soil property, which might not be used as one of the long-term cropland conditions in this study.

It is interesting to explore the dynamic of soil salinity over years and their effect on crop-drought vulnerability. We may investigate it in further work.

20. **Once again, salinity is more globally impacting drought-prone areas of the world**

Thank you for your point. We have incorporated the discussion of the effect of soil salinity associated with the discussion of soil acidity in the sentence. We have added some

possible mechanisms of soil salinity contribution exaggerating crop yield loss to drought. Please see the improved paragraph in the revised manuscript (see **Lines 281-288**).

21. AEI, A stands for Area?

    Yes, we have added “Area” in the sentence (**Line 306**).

22. Needless to state this obvious step

    We agree. We deleted the sentence(s) (**Line 311** and **Lines 325-326**).

23. You want to further investigate impact of temperature since you are linking drought to climate change (**Line 434**)

    Yes. We planned to investigate the impact of the extreme heat associated with climate change in further work.

24. Need to include the spatial zoning related to the regions of cultivation of these crops, no point in stating drought impact on rice, where rice is not cultivated for example!
    We have revised the conclusion following the reviewer's suggestion. We try to clarify the spatial zoning of the crops and drought impact, in regards to the type of crops as well. Please check them in the current version manuscript (see **Lines 443-446**).

Reviewer #2:

Have the authors made all data underlying the findings in their manuscript fully available? No

Regarding the availability of data underlying the analysis, we have provided them publicly available in the repository with DOI: **10.6084/m9.figshare.21298785**

The submitted manuscript investigated possible factors determining spatial pattern of drought impact on major crop yield sensitivity on a global scale

The overall framework of the study was well planned with enough information about the subject matter. Here are some specific comments with the hope that will help to clarify some things and improve the manuscript overall.

Thank you very much for your thorough check and comments to improve our manuscript. We have addressed all comments the reviewer has given.

1. The authors did not express their aim well enough in the last paragraph of the introduction section and should be written more clearly about how the results of the study will contribute to the literature.

This comment was similarly given by Reviewer #1. We have improved our introduction (last paragraph) by clarifying the goal and objectives of our study (see **Lines 82-93**).

2. Should be added to more literature related to this study in the introduction section.

We have enriched the literature referenced in the introduction section. You can check them throughout the introduction section.

3. Line 182 – line 183 and Line 196 – line 201 Authors should revise the results section. There seems to be discussing rather than pointing out the result of the study.

We have removed the sentence in **Lines 182-183** and **Line 196** as the reviewer suggested (Lines refer to the initial manuscript). However, several sentences in **Lines 197-201** are separated from the previous sentence which contains discussions. Therefore they might need to remain in the paragraph (see **Lines 218-222**).

4. Line 335 – line 347 Authors should rewrite and present them as results.

We really appreciate this suggestion. However, it seems that these parts can not be moved to the results sections. The reasons are that additional analysis made following the discussion on the possible cause of the irrigation parameter appears to be less important in the model. These additional analyses also are independent of the study framework. Therefore, we suggest not adding these parts to the results, otherwise, it may affect the coherence and flow of the study, we believe (**Lines 366-379**).

5. Authors should be more detailed about the data analysis session. The tool used for the analysis should be stated clearly with references.

We have indicated several tools used for analysis in the method sections (i.e., R, Python). Additionally, we have also mentioned the tool used to fit regression for obtaining crop-drought sensitivity as well (**Lines 162-164**).

Lines 162-164
These linear regression parameters are fitted based on the ordinary least-squares method using "statsmodels" library in Python [59]

Lines 173-174
Here, the Random Forest algorithm is applied using the "randomForest" library in R [62]

Lines 208-210
The map was made with "cartopy" library in Python (version 0.17) and the Natural Earth free vector and raster map data (naturalearthdata.com), which are freely available through the public domain [64]

6. Authors should make the results session come out clear and on point.

In general, we have improved the result section based on Reviewer' suggestions, alongwith the specific suggestions in regards to the result section that was also given by Reviewer #1.

7. Authors should improve the discussion section and avoid repetition of results in the discussion section. More literature should be included to make it solid.

Thank you for your point. Yes, we have made the discussion concise by removing unnecessary parts, especially in the first paragraph of the discussion section where we repeat mentioning some of the results.

In addition, we also added several extended references mentioned in the discussion section as reviewer suggested. Check them thorough the section.

8. Conclusion section should be shortened and concrete.

We have tried our best to shorten and make the conclusion as concise as possible in the current version. We appreciate your further suggestions.

9. The references section should be double-checked for the journal formatting style and rules.

We make sure that the manuscript has followed the formatting styles and rules used in PLOS ONE. We have made a substantial update in this current revised version of the manuscript. Thank you for all your comments and suggestions.

REFERENCES

Ivushkin, K., Bartholomeus, H., Bregt, A. K., Pulatov, A., Kempen, B., & De Sousa, L. (2019). Global mapping of soil salinity change. *Remote sensing of environment*, *231*, 111260.

Ribeiro, E., Batjes, N.H., Leenaars, J.G.B., van Oostrum, A.J.M., Mendes de Jesus, J. (2015). Towards the standardization and harmonization of world soil data: Procedures manual ISRIC World Soil Information Service (WoSIS version 2.0). Report 2015/03. ISRIC - World Soil Information, Wageningen, p. 100.
