# Supplementary material for: Possible factors determining global-scale patterns of crop yield sensitivity to drought
Source: PLoS One. 2023 Feb 2;18(2):e0281287. doi: 10.1371/journal.pone.0281287 (PMC9894396; doi:10.1371/journal.pone.0281287)
Supplement: Attachment — Submitted filename: PONE-D-22-24361R1_response-reviewers.pdf [file pone.0281287.s012.pdf]

Manuscript PONE-D-22-24361R1

Dear respected editor and reviewers,

Thank you for your feedback to improve our manuscript. This document responds to each comment and suggestion raised by the reviewer(s).

We have uploaded the revised manuscript with and without track changes.

A point-by-point response to the editor's comments

Reviewer #3

1. Revise the title in more meaningful style and avoid using many prepositions terms. Furthermore, consider the relation of your title with study contribution.
   Thank you for your suggestion. We have improved the title accordingly.

2. Related works section is missing therefore how authors can justify they have solve very problematic issue that not resolved by existing studies?
   We have discussed the existing studies in the previous version of our manuscript (mostly in Paragraphs 3 and 4). However, those might not be sufficient as reviewer suggested. Therefore, in the current revised version, we have added a paragraph discussing more specifically previous studies which are closely related to our study (see Paragraph 5, **Lines 83-96**).

3. What is the main gap of the study?
   According to our literature review shown in the current version of the introduction, we have mentioned the main gap of our study as follows:
   a. Quantitative identification of a wide range of factors across physical and socioeconomic factors that may determine crop-drought vulnerability patterns based on historical data and statistics.
      (discussed in Paragraph 2, **Lines 49-60**)
   b. The use of finer spatial scale data (grid-scale) may represent more heterogeneous agricultural systems smaller than those previous studies used (i.e., national and subnational level).
      (discussed in Paragraph 4, **Lines 74-82**)
   c. Discussion about key physical and socioeconomic factors in mitigating crop-drought vulnerability.
      (discussed in Paragraphs 3 and 5)

   We finally stated the objectives of the study in the last paragraph of our introduction for more clarity (**Lines 99-101**).

   Lines 99-101

"Particularly, this present study explores 1) how much global harvested area and crop yield anomaly are significantly affected by drought and 2) how the key factors mitigate or exacerbate the crop-drought vulnerability in terms of their crop yield sensitivity to drought."

4. The contribution of the work is not clear for me there is no new model has presented. The contribution is basically is analysis several factors. Therefore, authors are suggested to highlight the contributions of the study in term of list at the end of related work section.
We consider reviewer's suggestion by highlighting the contribution of our study in the conclusion section. We hope this can be sufficient to make the reader understand what our study wants to deliver (see **Lines 471-477**).

Lines 471-477
"Our study also reveals how such important factors affect crop-drought vulnerability: 1) crop yield in semi-arid and arid regions with low annual precipitation and higher PET is more sensitive to drought impact; 2) other important factors such as higher fertilizer rate, elevation, soil water holding capacity and shorter growing season have a moderate association with lower vulnerability, 3) the effect of other socioeconomic indicators (i.e., GDP per capita, irrigation) may be underestimated in our models generally due to limitation in modeling the actual mechanism by the selected proxies based the currently available global dataset."

5. Since authors used machine learning model for their analysis. An evaluation process should be conducted to check efficiency of used model for instance mean square error value.
We have added the more goodness of fit indicator RMSE (Root Mean Square Error) in the paragraph in addition to the R-square (see **Lines 246-252**).

6. Another question why author used random forest model? Why not another model?
Random forest, one of the ensemble learning in machine learning, is used in this study. This selection is based on previous studies, which reported that this model has more accuracy and robustness than any common machine learning method (e.g., decision trees, gradient boosting) (Breiman, 2001; Park et al., 2016). The random forest also was reported to run efficiently on a large dataset and feature variables and is not sensitive to noise or over-fitting (Breiman, 2001; Jo & Cheng, 2014). This model is simpler in implementation as it has fewer input parameters than other machine-learning algorithms.
We have added these particular explanations in the revised manuscript (see **Lines 183-184**)

Lines 183-184
"We use Random Forest algorithm due to its relatively better accuracy, interpretability, and efficiency with large training datasets than other machine learning methods (e.g., decision trees, nearest neighbors) [62–64]."

7. Also the results from random forest should be compared with other machine learning model to show for the reader how far the selected model is better.
We acknowledge the reviewer's concern. Therefore, we have made a substantial improvement in our manuscript by making an additional analysis. We tested the model robustness by comparing Random Forest model with other two machine learning algorithms: 1) Support Vector Regression, and 2) Extreme Gradient Boosting. Generally results show that our models are robust across different machine learning models.
We added these explanations and results in the following paragraphs:

Lines 184-188

"In addition, we test the sensitivity of our results by comparing the model performances estimating crop-drought sensitivity with the outputs of two other machine learning algorithms: support vector regression (SVR) [65] and gradient boosting (XGBoost) [66], implemented in the R library, "caret" [67]...."

Lines 240-243

"The explanatory skills of the 22 variables are robust across different types of machine learning algorithms (SVR and XGBoost), showing similar model performances with a small standard deviation of R2 and RMSE by 1% and 0.2%, respectively, across crop types and machine learning algorithms (S4 Table)."

Lines 282-282

"We also obtain the partial dependence plots based on SVR and XGBoost (S7 and S8 Figs). The results confirm that most estimated relationships between various determinants and crop yield sensitivity are generally robust across the models."

References

Breiman, L., 2001. Random forests. *Machine learning*, *45*(1), pp.5-32.
Park, S., Im, J., Jang, E. and Rhee, J., 2016. Drought assessment and monitoring through blending of multi-sensor indices using machine learning approaches for different climate regions. *Agricultural and forest meteorology*, *216*, pp.157-169.
Jo, T. and Cheng, J., 2014, December. Improving protein fold recognition by random forest. In *BMC bioinformatics* (Vol. 15, No. 11, pp. 1-7). BioMed Central.
