# Supplementary material for: Possible factors determining global-scale patterns of crop yield sensitivity to drought
Source: PLoS One. 2023 Feb 2;18(2):e0281287. doi: 10.1371/journal.pone.0281287 (PMC9894396; doi:10.1371/journal.pone.0281287)
Supplement: Attachment — Submitted filename: PLOS_PONE-D-22-24361_reviewer.docx [file pone.0281287.s009.docx]

PLOS ONE

Possible factors determining spatial patterns of drought impact on major crop yield sensitivity on a global scale

--Manuscript Draft--

| Manuscript Number: | PONE-D-22-24361 |
| --- | --- |
| Article Type: | Research Article |
| Full Title: | Possible factors determining spatial patterns of drought impact on major crop yield sensitivity on a global scale |
| Short Title: | Possible factors determining spatial patterns of drought impact on major crop yield sensitivity |
| Corresponding Author: | Vempi Satriya Adi Hendrawan  Tohoku University Graduate School of Environmental Studies: Tohoku Daigaku Daigakuin Kankyo Kagaku Kenkyuka  Sendai, JAPAN |
| Keywords: | drought yield sensitivity; global crop-drought relationship; random forest model; standardized precipitation index |
| Abstract: | In recent years, droughts have critically limited crop production, inducing food system shocks in a region and globally. Previous studies estimated that crop yield variability in around one-third to three-fourths of global harvested areas is explained significantly by drought, revealing the notable vulnerability of crop systems to such climate stressors. However, understanding the key factors determining the global pattern of crop yield sensitivity to drought is limited. Here, we investigate a wide range of physical and socioeconomic factors that may determine the sensitivity of global crop yield to drought based on the Standardized Precipitation Index at 0.5° resolution from 1981 to 2016 using a machine learning approach. The results indicate that the spatial variations of the crop-drought sensitivity are mainly explained by environmental factors (i.e., annual precipitation, soil water-holding capacity, soil acidity, annual potential  evapotranspiration) and management factors (i.e., fertilizer application rate, growing season). Our study may underestimate the influence of socioeconomic and irrigation parameters due to the study approach and data limitations. Nevertheless, this study provides the first attempt to understand global crop-drought sensitivity patterns and their possible determinants. This understanding may provide insights for further studies addressing better crop-drought vulnerability measures under future stress. |
| Order of Authors: | Vempi Satriya Adi Hendrawan |
|  | Daisuke Komori |
|  | Wonsik Kim |
| Additional Information: |  |
| Question | Response |
| Financial Disclosure  Enter a financial disclosure statement that describes the sources of funding for the work included in this submission. Review the [submission guidelines](http://journals.plos.org/plosone/s/submission-guidelines#loc-financial-disclosure-statement) for detailed requirements. View published research articles from [PLOS ONE](http://journals.plos.org/plosone/) for specific examples.  This statement is required for submission and will appear in the published article if the submission is accepted. Please make | D.K. was partly supported by the International Joint Graduate Program in Resilience and Safety Studies, Tohoku University. The funder had no role in study design, data collection and analysis, decision to publish, or preparation of the manuscript. |

| sure it is accurate. |  |
| --- | --- |
| Unfunded studies Enter: The author(s) received no specific funding for this work. Funded studies Enter a statement with the following details: - Initials of the authors who received each award - Grant numbers awarded to each author - The full name of each funder - URL of each funder website - Did the sponsors or funders play any role in the study design, data collection and analysis, decision to publish, or preparation of the manuscript? - NO - Include this sentence at the end of your statement: The funders had no role in study design, data collection and analysis, decision to publish, or preparation of the manuscript. - YES - Specify the role(s) played. |  |
| * typeset |  |
| Competing Interests  Use the instructions below to enter a competing interest statement for this submission. On behalf of all authors, disclose any [competing interests](http://journals.plos.org/plosone/s/competing-interests) that could be perceived to bias this work—acknowledging all financial support and any other relevant financial or nonfinancial competing interests.  This statement is required for submission and will appear in the published article if the submission is accepted. Please make sure it is accurate and that any funding sources listed in your Funding Information later in the submission form are also declared in your Financial Disclosure statement.  View published research articles from [PLOS ONE](http://journals.plos.org/plosone/) for specific examples. | The authors have declared that no competing interests exist. |

| NO authors have competing interests Enter: The authors have declared that no competing interests exist. Authors with competing interests Enter competing interest details beginning with this statement: I have read the journal's policy and the authors of this manuscript have the following competing interests: [insert competing interests here] |  |
| --- | --- |
| * typeset |  |
| Ethics Statement  Enter an ethics statement for this submission. This statement is required if the study involved:   - Human participants - Human specimens or tissue - Vertebrate animals or cephalopods - Vertebrate embryos or tissues - Field research   Write "N/A" if the submission does not require an ethics statement.  General guidance is provided below. Consult the [submission guidelines](http://journals.plos.org/plosone/s/submission-guidelines#loc-guidelines-for-specific-study-types) for detailed instructions. Make sure that all information entered here is included in the Methods section of the manuscript. | N/A |

Format for specific study types

Human Subject Research (involving human

participants and/or tissue)

Give the name of the institutional review

board or ethics committee that approved the

study

•

Include the approval number and/or a

statement indicating approval of this

research

•

Indicate the form of consent obtained

(

written/oral) or the reason that consent was

not obtained (e.g. the data were analyzed

anonymously)

•

Animal Research (involving vertebrate

animals, embryos or tissues)

Provide the name of the Institutional Animal

Care and Use Committee (IACUC) or other

relevant ethics board that reviewed the

study protocol, and indicate whether they

approved this research or granted a formal

waiver of ethical approval

•

Include an approval number if one was

obtained

•

If the study involved

non-human primates

,

add

additional details

about animal welfare

and steps taken to ameliorate suffering

•

If anesthesia, euthanasia, or any kind of

animal sacrifice is part of the study, include

briefly which substances and/or methods

were applied

•

Field Research

Include the following details if this study

involves the collection of plant, animal, or

other materials from a natural setting:

Field permit number

•

Name of the institution or relevant body that

granted permission

•

Data Availability

Authors are required to make all data

underlying the findings described fully

available, without restriction, and from the

time of publication. PLOS allows rare

exceptions to address legal and ethical

concerns. See the

[PLOS Data Polic](http://journals.plos.org/plosone/s/data-availability)

[y](http://journals.plos.org/plosone/s/data-availability)

and

[FA](http://journals.plos.org/plosone/s/data-availability#loc-faqs-for-data-policy)

[Q](http://journals.plos.org/plosone/s/data-availability#loc-faqs-for-data-policy)

for detailed information.

Yes - all data are fully available without restriction

| A Data Availability Statement describing where the data can be found is required at submission. Your answers to this question constitute the Data Availability Statement and will be published in the article, if accepted. |  |
| --- | --- |
| Important: Stating ‘data available on request from the author’ is not sufficient. If your data are only available upon request, select ‘No’ for the first question and explain your exceptional situation in the text box. |  |
| Do the authors confirm that all data underlying the findings described in their manuscript are fully available without restriction? |  |
| Describe where the data may be found in full sentences. If you are copying our sample text, replace any instances of XXX with the appropriate details.   - If the data are held or will be held in a public repository, include URLs, accession numbers or DOIs. If this information will only be available after acceptance, indicate this by ticking the box below. For example: All XXX files   are available from the XXX database (accession number(s) XXX, XXX.).   - If the data are all contained within the manuscript and/or Supporting Information files, enter the following: All relevant data are within the manuscript and its Supporting Information files. - If neither of these applies but you are able to provide details of access elsewhere, with or without limitations, please do so. For example:   Data cannot be shared publicly because of [XXX]. Data are available from the XXX Institutional Data Access / Ethics Committee (contact via XXX) for researchers who meet the criteria for access to confidential data.  The data underlying the results presented in the study are available from (include the name of the third party | Source codes of the analysis and the datasets generated during and/or analyzed during the current study are available from the corresponding authors upon request. |

| and contact information or URL).  • This text is appropriate if the data are owned by a third party and authors do not have permission to share the data.  * typeset |  |
| --- | --- |
| Additional data availability information: | Tick here if your circumstances are not covered by the questions above and you need the journal’s help to make your data available. |

Manuscript	[Click here to access/download;Manuscript;PLOS_article_r2-](https://www.editorialmanager.com/pone/download.aspx?id=31772667&guid=a7e9cd75-edab-4815-b484-583debd03650&scheme=1)

[reviewer.docx](https://www.editorialmanager.com/pone/download.aspx?id=31772667&guid=a7e9cd75-edab-4815-b484-583debd03650&scheme=1)

# PLOS ONE

1 **Possible factors determining spatial patterns of drought**
2 **impact on major crop yield sensitivity on a global scale**
3 **Vempi Satriya Adi Hendrawan**^1,2,*^**, Daisuke Komori**^1,3,*^**, Wonsik Kim**^4^
4 ^1^ Graduate School of Environmental Studies, Tohoku University, Sendai, 980-8579, Japan
5 ^2^ Department of Civil and Environmental Engineering, Universitas Gadjah Mada, Yogyakarta, 55281, 6 Indonesia
7 ^3^ Graduate School of Engineering, Tohoku University, Sendai 980-8579, Japan
8^4^ Institute for Agro-Environmental Sciences, National Agriculture and Food Research Organization, 9	Tsukuba 305-8604, Japan

10

11 * Corresponding author
12 E-mail: vempi@dc.tohoku.ac.jp (VSAH), daisuke.komori.e8@tohoku.ac.jp (DK)

# 13 Abstract

14 In recent years, droughts have critically limited crop production, inducing food system shocks in a
15 region and globally. Previous studies estimated that crop yield variability in around one-third to three-
16 fourths of global harvested areas is explained significantly by drought, revealing the notable
17 vulnerability of crop systems to such climate stressors. However, understanding the key factors
18 determining the global pattern of crop yield sensitivity to drought is limited. Here, we investigate a
19 wide range of physical and socioeconomic factors that may determine the sensitivity of global crop
20 yield to drought based on the Standardized Precipitation Index at 0.5° resolution from 1981 to 2016
21 using a machine learning approach. The results indicate that the spatial variations of the crop-drought

sensitivity are mainly explained by environmental factors (i.e., annual precipitation, soil water-holding capacity, soil acidity, annual potential evapotranspiration pattern) and crop management factors (i.e., fertilizer application rate, growing season pattern). Our study may underestimate the influence of socioeconomic and irrigation parameters due to the study approach and data limitations. Nevertheless, this study provides the first attempt to understand global crop-drought sensitivity patterns and their possible determinants. This understanding may provide insights for further studies addressing better crop-drought vulnerability measures under future stress.

**Keywords:** drought yield sensitivity, global crop-drought relationship, random forest model, standardized precipitation index

# 1. Introduction

The impact of global climate variabilities on crops has been well investigated. Empirically, around one-third of the variability of crop measures (i.e., crop yield, production) (1–3) and harvested area globally (4) are explained by climate variabilities during the current period. As one climate extreme, drought has been widely recognized as a notable limiting factor for crop yield and production. One-third to three-fourths of the global harvested areas experienced yield losses due to drought (5,6), corresponding to approximately 166 billion US dollars in the current period (5). The serious drought impact on crops may unveil the global crop system's vulnerability to the climate stressor, which can later threaten local or global food security in the current and future climate (3,7,8).

Approaches for understanding the drought risk on the crop system may include assessing its potential impact and crop vulnerability under specific dry condition definitions (e.g., precipitation deficit, soil moisture stress) (9–11) during the growing period as a hazard and exposure measures (7). The drought impact on crops can be measured by damages or losses on crop production indicators (i.e., crop yield, crop production, or harvested area) due to certain drought conditions, whereas the vulnerability can be measured by the sensitivity or susceptibility of the crop production responding a drought severity (12). While the drought impact on crops and their vulnerability is growingly evident (through field-scale experiments to larger scale based on global physical modeling and statistics (1,13–15)), quantitative identification of multiple factors that may determine such crop-drought relationship patterns across global agricultural systems is limited in previous works. Indeed, these crop-climate relationships can be highly associated with other socio-economic and management factors as a whole system.

Agricultural risk and vulnerability to drought in a region may be determined by several factors (12), which can be generally grouped as physical (e.g., climate, soil, topography, crop production factors) and socio-economic (e.g., management, fertilizer input, field size, demography, GDP per capita, technology) (16–18). These determinants govern the adaptive capacity of a system to climate disruptions over time. For instance, studies (5,19) reported that technological improvements (i.e., high yield breeding, adoption of drought-tolerant seed) associated with per capita GDP growth could further increase the resilience of crop production systems to droughts in a region. Current reports also revealed that more resilient agricultural systems could adapt to stress and recover better than those less resilient, even with a comparable or higher climate extreme exposure (17,19). Therefore, quantifying the adverse impact on crops due to extreme events such as a drought is imperative to understanding crop vulnerability to climate disruptions since drought impact is known to be one of the most disastrous climate extremes causing crop damage.

Furthermore, how socio-economic factors drive the global crop-drought relationship is often overlooked. Several studies (10,16,18) attempted to quantitatively explore how various physical and socio-economic factors might control crop productivity measures (i.e., crop yield, production). Even though numerous existing local studies with a specific context and underlying physical and socioeconomic conditions existed (20,21), these studies might not represent the understanding to a larger extent. On a global scale, previous studies often use aggregated country or sub-country level data to assess socio-economic factors’ influence to crop vulnerability (16,17,22,23). This approach might not represent heterogeneous agricultural systems, especially within a large administrative size, given that adaptive capacity may differ even within local or smaller levels (24).

This study addresses the above gaps by employing statistical models based on gridded global historical crop yield, precipitation, and various datasets. We examine the sensitivity of maize, rice, soybean, and wheat yield to drought based on a meteorological drought indicator (Standardized Precipitation Index, SPI) with a spatial resolution of 0.5° grid-cell. This study presents the first attempt to explore the key factors in determining the global crop-drought sensitivity across various physical and socio-economic variables based on historical data on a gridded scale. This study may provide light for further attempts to understand crop system vulnerability and its determinants for future adaptation and mitigation efforts in the global context.

# 2. Materials

The dataset used in this study mainly consists of global gridded crop yield data, Standardized Precipitation Index (SPI), and several indicators from various sources that possibly control crop sensitivity to drought. We consider all the data from 1981 to 2016 associated with this study analysis period. Since the spatial resolution of the datasets varies, we re-gridded all the datasets to 0.5° using bilinear interpolation.

## 2.1. Drought Indicators

Here, the drought indicator is based on one of the meteorological drought indices, the Standardized Precipitation Index (SPI) (25). While SPI is solely based on precipitation, this index has been widely used and well recognized in previous drought studies and applications (26–29). Other drought indexes such as PDSI, SPEI, and other indices (e.g., soil moisture anomaly) have also been used but require more parameterization, which may introduce additional uncertainties (30,31). Therefore, the simple but robust drought definition based on SPI is employed in this study to reveal the global drought pattern from 1981-2016.

SPI is obtained based on the standardization of monthly precipitation accumulation within various timescales (e.g., 3, 6, 9, 12 months) over the window period of 1981-2016; thus, the standardized value finally has a mean of zero and a standard deviation of one. Before standardization based on the normal Gaussian distribution, the data is fitted based on the Gamma distribution (32). Here, SPI is developed based on several historical global gridded precipitations to obtain the best agreement among datasets according to their ensemble mean (GPCC, CRU, PRECL, UDEL, CPC, MSWEP, MERRA-2, and

ERA-5 dataset) (6). SPI is produced for each dataset, and then the mean of the eight selected datasets is obtained to represent ensemble SPI as the drought index.

The drought index (𝐷𝐼) based on negative SPI is used to represent the inter-annual drought condition in this study (**Eq 1)** (6). We obtain yearly 𝐷𝐼 based on monthly SPI on harvest month each year. The harvest month data for each grid cell is obtained from the crop calendar dataset during the year 2000 (33) and is constantly applied every year from 1981-2016. The drought index (𝐷𝐼) is calculated as:

 (1)

where 𝑆𝑃𝐼_𝑚,𝑡_ is the 9-month SPI (SPI-9) in harvest month 𝑚 and year 𝑡. SPI with a 9-month timescale is used to indicate a long-term precipitation anomaly which may indicate a more prominent drought

(25,26). The spatial variation of the global crop-drought relationship is broadly similar when different SPI timescales are used (6).

## 2.2. Crop yield anomaly

The percentage of yield anomaly (Δ𝑌) is estimated based on the gridded dataset of historical yield (GDHY) (34) for the major crops: maize, rice, soybean, and wheat (**Eq 2**). We calculate long-term crop yield trends as normal or expected yields (𝑌̅) using the local polynomial regression method (6). This method is selected since it can account for possible non-linear trends and works well for limited data series, particularly compared to the moving average method (35). This long-term trend generally represents the improvement of technological advances in producing higher yields in a region, while the yield anomaly subtracted from the expected yield is assumed to be caused by climate-related factors, although other affecting factors, such as socioeconomic factors, may also influence this short-term anomaly.

 (2)

where 𝑌 is crop yield (t ha^-1^) and 𝑌̅ is the trend obtained by local polynomial regression (t ha^-1^) in a given year 𝑡.

## 2.3. Crop-drought determinants

We obtained several possible factors determining crop yield and drought relationship. We defined the possible determinants as (1) climate factors (1), (2) terrain factors (36,37), (3) soil factors (38,39), (4) irrigation factors (22,23), (5) crop production (hereafter production) factors (40), (6) fertilizer factors (17,41), and (7) socio-economic factors (10) (see **Table 1**). All variables shown in **Table 1** are then used as independent variables to train the Random Forest model (see Methods). Moreover, it is noteworthy that we used an average value or a subset of the dataset in a specific year between 1981-2016, despite a possible change during the study period. Previous studies (e.g., 39) have also used this approach, and year-specific datasets are widely employed, especially in the case of crop calendar and harvest area datasets (42,43), which were only available around the year 2000.

136

137	**Table 1**. Determinants used to describe the crop-drought sensitivity analyzed in the study.

| Class | Variable | Unit | Period | Source |
| --- | --- | --- | --- | --- |
| Climate | Mean annual precipitation | mm year^-1^ | 1981 –  2016 | (44) |
|  | Mean annual PET | mm year^-1^ |  |  |
|  | Mean temperature | °C |  |  |
| Terrain | Elevation | meter | 2000 | (45) |
|  | Slope | - |  |  |
| Soil | Topsoil saturated hydraulic conductivity | cm s ¹ | 2013 | (46) |
|  | Topsoil clay amount | % weight | 2000 | (47) |
|  | Topsoil organic carbon | kg C m^-2^ |  |  |
|  | Topsoil acidity | pH |  |  |
|  | Water-holding capacity | mm | 1993 | (48) |
| Irrigation | Area equipped for irrigation (AEI) | % of land area | 2005 | (43,49) |
|  | Area actually irrigated (AAI) | % of AEI |  |  |
|  | Area irrigated with groundwater |  |  |  |
|  | (AEIGW) | % of AEI |  |  |
|  | Area irrigated with water from nonconventional sources (AEINC) | % of AEI |  |  |

| Production | Growing season length | days | The 1990s  – early  2000s | (33) |
| --- | --- | --- | --- | --- |
|  | Harvested area | km² year^-1^ | 1998 –  2002 | (42) |
| Fertilizer | Nitrogen rate application | kg ha^-1^ | 2000 | (50) |
|  | Phosphorus rate application | kg ha^-1^ |  |  |
|  | Potassium rate application | kg ha^-1^ |  |  |
| Socioeconomic | GDP | Billion USD | 2015 | (51) |
|  | GDP per capita | USD |  |  |
|  | Population | People | 2015 | (52) |

# 3. Methods

## 3.1. Crop-drought sensitivity

We estimate crop-drought sensitivity as the slope coefficient (𝛽) of a linear regression between yield anomaly (Δ𝑌) and 𝐷𝐼 in the given year as time series (**Eq 3**). Statistical significance considered in this study is based on a two-tailed t-test with a threshold of 𝑃 < 0.05.

Δ𝑌 = 𝑎 + 𝛽 · 𝐷𝐼	(3)

where 𝑎 and 𝛽 are the model intercept and slope, respectively. These linear regression parameters are fitted based on the least-squares method (see **S1 Fig** for an example of the regression model in a grid cell). This calculation is applied for each grid cell and crop. Here, crop-drought sensitivity refers to the simple linear relationship, despite possible non-linear responses and more complex relationships occurring across regions. Nevertheless, this simple model allows us to understand the general pattern of how crop yield responds to drought, as demonstrated by previous studies (1,53).

## 3.2. Random forest

Here we use one of the machine learning algorithms, Random Forest (54), to reveal the key factors defining the crop-drought relationship. We set the crop-drought sensitivity (*β*) as the response variable and various possible determinants (**Table 1**) as independent variables or predictors. The Random Forest model has been used to detect a non-linear climate-crop system relationship (2,39). Here, the Random Forest algorithm is applied using the “randomForest” library in R (55). We used parameter input as default settings (e.g., number of trees = 500) since the model skill with basic setting is adequate to reveal the variable importance and their influence on global crop sensitivity (39). The model is fitted with the data from all available grid cells (representing spatial dimension) for each crop. During the calculation process, explained variance is calculated based on “out-of-bag” error estimation by

subsampling with replacement in the training samples (55).

Further, we obtain relative importance (56) for each variable based on the fitted models. Here we use the metric measuring number of tree nodes employing the variable to reduce the model’s impurity averaged across all trees (55). The “randomForest” calculates and scales it as the total importance for all variables equals one. Then, for ease of comparison, we normalized them into the 0-1 range, representing the minimum and maximum values, respectively. Finally, we plot all models based on different settings and rank them from the most important parameter to the least.

We use a partial dependence plot from the fitted models to understand each variable’s relationship with the response variable (56). The partial dependence allows selecting small subsets or single variables to reveal a relationship between predictor(s) and a response variable, similar to sensitivity analysis (2). Here, we picked up the six most influential variables based on their overall relative importance (from the average of all experiment setups) to demonstrate their relationship with the response variable. This enables us to estimate the effect of the important variable to determine global crop sensitivity to drought.

# 4. Results

## 4.1. Crop-drought sensitivity pattern

We obtain crop-drought sensitivity based on the slope of the linear regression (𝛽) between crop yield anomalies and the meteorological Drought Index (𝐷𝐼) based on SPI with a 9-month timescale (**Fig 1**). Significant relationships are evident in around a quarter of croplands or over around one-fourth of global maize, wheat, and soybean croplands, respectively (𝑃 < 0.05). Almost entire significant croplands show a negative correlation (~96%), confirming the damaging impact of drought on major crops. In contrast, the negative impact of drought on rice seems offset by yield benefit and remains low globally, with only 7% significantly correlated croplands. These results agree with previous studies using the drought magnitude (5).

**Fig 1. Crop yield sensitivity to drought based on 9-month SPI across global croplands based on GDHY for maize, rice, soybean, and wheat.** Sensitivity is calculated by the linear slope coefficient (*β*) as crop yield anomaly (%) per drought magnitude based on SPI, while white cells show no cropland or crop yield data. We show a map over entire global croplands for a spatially broader interpretation. The map presents only areas with significant crop yield sensitivity to drought (𝑃 <

0.05) is provided in **S2 Fig**.

Globally, drought reduces yield by 4%, 1%, 3%, and 6% for maize, rice, soybean, and wheat, respectively, with increasing drought index 𝐷𝐼 (i.e., precipitation deviations from normal condition per precipitation standard deviations). Over a quarter of the total croplands with significant drought impact, yields drop by around 14% by one drought unit (maize, soybean, and wheat). Furthermore, we point out primary regions with a substantial drought impact, comprising generally dry croplands such as The Great Plains, Africa (southern Africa, The Sahel, and the Horn of Africa), Australia, northeast China, the Mediterranean basin, and Central Asia (**Fig 1**), confirming the previous findings (5,6). Droughts also slightly damage maize and wheat yield in wetter regions such as South America, The Pampas, and the African and Southeast Asian tropics. While droughts induce rice yield loss in parts of The Sahel region and Northeast China and slightly in central Asia, rice in the tropics broadly shows weak sensitivity to drought. The higher crop yield sensitivity over drylands may indicate their higher vulnerability along with higher drought hazard exposures.

## 4.2. Possible driving factors

We investigate the importance of several possible determinants as independent variables to control crop-drought sensitivity. We train the Random Forest models for regression with predictors of 22 variables. Results show that the variable predictors can explain, on average, 43%, 36%, 66%, and 37% of crop-drought sensitivity variations for maize, rice, soybean, and wheat, respectively. It is worth noting that models for rice show lower skill, which may be due to the fewer variations of crop-drought sensitivity over the available grid cells globally.

Here, we derive each variable’s relative contribution to the models. While the important parameters vary across crops, we average their relative importance to reveal overall important variables (**Fig 2)**. It shows that mean annual precipitation, water-holding capacity, soil acidity, Potential Evapotranspiration (PET), elevation, and potassium application rate are among the most driving factors, revealing that climate, soil, terrain, and agricultural input factors play an important role in crop-drought sensitivity variations. In particular, for each crop, the result suggests that the annual precipitation pattern becomes important for all crops, while water-holding capacity, elevation, PET, acidity, and potassium rate are relevant for some crops (**Table 2**). Regarding management factors, groundwater irrigation potential and population are slightly important for maize, soybean, and rice.

**Fig 2. The relative importance of the determinant factors in explaining crop-drought sensitivity.** The importance values are normalized to the 0-1 range computed separately across different crops. The colored bars represent the average importance. Label order of the y-axis indicates the order from the most important variables to the least based on the overall average across crops.

**Table 2**. Top six important parameters for each crop based on the average relative importance. The overall average represents the average from combinations across crops corresponding to the order of the y-axis labels in **Fig 2.**

| Ran  k | Maize | Rice | Soybean | Wheat | Overall average |
| --- | --- | --- | --- | --- | --- |
| 1 | Precipitation | Precipitation | Water-holding capacity | Precipitation | Precipitation |
| 2 | Water-holding capacity | PET | Potassium rate | Acidity | Water-holding capacity |
| 3 | Elevation | Population | Irrigation  (groundwater) | Water-holding capacity | Acidity |
| 4 | Growing season length | Acidity | Precipitation | Nitrogen rate | PET |
| 5 | Irrigation  (groundwater) | Elevation | Elevation | Hydraulic conductivity | Elevation |
| 6 | Soil organic carbon | Irrigation  (groundwater) | PET | Temperature | Potassium rate |

## 4.3. Impact behavior of each factor

We obtained a partial dependence plot for each variable to describe their functional relationship with crop-drought sensitivity patterns. The subsequent sections discuss the variable relationship for each parameter among different factors (climate, terrain, soil, production, irrigation, fertilizer, and socioeconomic factors).

### 4.3.1. Climate factors

The most important factor, mean annual precipitation, demonstrates a clear relationship with crop drought sensitivity; a higher precipitation rate alleviates crop yield reduction due to drought. For instance, based on functional relationships in maize and wheat in which the yield loss sensitivity is high, the increase of the higher annual precipitation (e.g., ~500-1000 mm year^-1^) may mitigate yield loss (**Fig 3**). This result indicates that crop yield in semi-arid and arid regions with less precipitation is more sensitive to drought impact, as reported elsewhere (5,57–59). Furthermore, this confirms that crop loss sensitivity increases in most drylands, where severe hazards and higher vulnerability occur concurrently. The other climate factor, PET, shows an inverse response to precipitation—higher PET tends to aggravate yield loss sensitivity due to drought, while temperature slightly determines the response variables.

### 4.3.2. Terrain factors

The higher elevation is found to be an important parameter with a diminishing effect on yield loss sensitivity. The response is particularly evident for maize, soybean, and wheat. This implies that more serious crop-yield loss sensitivity due to drought is generally located in low-lying cropland; while in higher elevations, where precipitation rate is generally higher, drought impact may be lessened (60). Moreover, temperature and saturated water vapor would generally be lower at higher altitudes, thus lowering atmospheric vapor demand that may alleviate moisture stress due to drought (61). On the other hand, the other terrain factor, the slope, does not show a clear relationship with the drought-induced yield sensitivity.

### 4.3.3. Soil factors

The most important soil factor, water-holding capacity, generally shows a consistent pattern for all crops. The higher value may slightly moderate drought-induced yield loss, as indicated in the previous studies (62,63). For instance, this parameter's functional relationship shows a decrease in drought-induced yield loss in all crop regions by around 330 cm water per cm water-holding capacity. The other soil factor, soil acidity, tends to increase the drought-induced yield loss by less acidic (alkaline) soil, especially for soybean and wheat. This response may also be controlled by the general relationship between precipitation regime and soil acidity —alkaline soils are often found in arid and semi-arid regions (64). Moreover, hydraulic conductivity may also lessen drought-induced yield loss, especially for maize and wheat within hydraulic conductivity values (i.e., up to 25 cm s^-1^). This dependence generally may be related to more effective water transport and increasing soil water content potential as soil hydraulic conductivity increases (65,66), despite more complex mechanisms that may be involved (13,67). The other parameters: soil organic carbon and clay soil amount, however, generally remain less influence given lower relative importance in the model, despite their importance in some cases (e.g., higher clay soil amount in wheat seems to slightly aggravate negative crop sensitivity to drought).

### 4.3.4. Production factors

The crop production factor, growing season length, is revealed as one of the important factors that may worsen wheat yield loss due to longer season length, particularly for maize and wheat. The longer season length is generally attributable to winter wheat being dominated by a global rainfed system, and the crop may be highly reliant on precipitation and sensitive to precipitation variability. For instance, the longer maize growing season (>~130 days) may exacerbate the yield loss. This growing season covers most of the major maize belts in higher latitudes, including most semi-arid and arid lands, while the shorter season spans around the tropics. The other parameter, the harvested area, may exhibit lesser effects.

### 4.3.5. Irrigation factors

Overall, irrigation parameters (i.e., percentage of equipped for irrigation, AEI; actual irrigation, groundwater; and non-conventional water sources) show an unclear relationship with the yield sensitivity, despite a slightly higher relative importance, especially for the groundwater irrigation parameter. The remaining irrigation parameters tend to have less control over the drought-induced yield sensitivity. We acknowledge that these results contrast with previous studies arguing that irrigation may partly mitigate drought impact to crop yield. ~~We further discuss this point in~~ **~~Discussion~~**~~.~~

### 4.3.6. Fertilizer factors

The fertilizer factors, potassium and phosphorus application rates, are revealed among the important variables. In general, their higher application rate may slightly alleviate yield loss due to drought. These fertilizer applications may effectively relieve drought stress on crops and enhance drought tolerance (68–70). This mechanism may be generally explained by reducing the uptake of toxic nutrients and strengthening the physiological efficiency (68), which may improve overall resilience to drought, while in the particular further mechanism of the role of nutrients to mitigate drought stress on plants has been discussed in previous studies (71). Moreover, the other fertilizer factor, nitrogen application rate, does not show an apparent relationship with the yield sensitivity indicator, despite a notable exception for the wheat with an easing effect on the drought-induced yield loss.

### 4.3.7. Socio-economic factors

GDP per capita relatively shows a stronger relationship with the yield sensitivity than the GDP parameter, especially in the case of wheat and soybean. However, these relationships remain weak, preventing us from drawing a conclusion. ~~We further discuss these factors' importance in defining crop sensitivity to drought in~~ **~~Discussion~~**~~.~~ The other factor, the population indicator, does not determine yield sensitivity to drought.

**Fig 3. Relationship between the response variable of crop-drought sensitivity (****) as the response variable and the six selected determinant factors as independent variables based on each partial dependence using Random Forest models.** The order shows higher to lower overall relative importance across crops (see **Table 2**). The lower negative y-axis indicates higher yield loss due to drought. The partial dependence plot for all variables (22 variables) is provided in **S3 Fig**.

# 5. Discussion

The present study assesses crop sensitivity to drought from 1981 to 2016 based on a meteorological drought index using a 9-month Standardized Precipitation Index (SPI) and gridded crop yield datasets for maize, rice, soybean, and wheat. Our estimates reveal that over one-fourth of global cropland is significantly sensitive to inter-annual drought, which generally agrees with previous studies (5,59), despite slightly lower estimates than the previous study using a similar approach and dataset (5). A negligible drought impact on rice is also confirmed in this study, which may be attributable to its low exposure to drought with general moisture surplus as reported elsewhere (53,72). Here, we highlight a corresponding result pattern among related studies based on specific drought indicators and crop statistics during historical periods (5,73,74). Regionally, we find that the dryland Mediterranean (Europe), Southern Africa, South-eastern South America, Midwestern North America, Northern Asia, and Australia are the hotspots of the drought-sensitive regions, particularly for the three most affected crops: maize, soybean, and wheat (see **S3 Fig**). The significance of these regions is also widely highlighted by previous studies (5,9,59).

This study explores several possible factors determining crop sensitivity to drought. We indicate that environmental factors (i.e., climate, soil, terrain) have an important role in mitigating crop yield loss due to drought, while management factors such as fertilizer, growing season, and GDP per capita show moderate influence, while the irrigation factor seems to have lesser control, in contrast to their typical role in mitigating the impact of drought (2,23,53,72,75). We discuss these findings regarding the irrigation effect as follows. First, we may not detect the significant importance of irrigation factors as we cannot independently disentangle the impact of drought on irrigated and rainfed crop yields. Here, we use mean annual yield involving multiple crop growing systems (irrigated and rainfed) and seasons (major and minor) (34). Therefore, our estimates may offset the mitigating effect of irrigation due to a comparable or even larger negative impact of drought on rainfed crops within the same grid location. Previous studies primarily conducted on a local or regional scale employed irrigated and rainfed yield data separately to obtain drought impact on each system (75), while further challenges related to data availability may arise when dealing with a global scale analysis.

On the other hand, another study (2) detected response of inter-annual yield using sub-country-level data (combined rainfed and irrigated yields) on each classified irrigated and rainfed cropland based on a specific definition (e.g., irrigated cropland is defined as >80% of harvested area irrigated and otherwise is rainfed). Similarly, we classify irrigated and rainfed cropland based on the similar global harvested area dataset (42) and the classification used in the previous study (2). We then calculate the share of drought-affected areas for each crop-drought sensitivity bin (**Fig 4**). We find that the rainfed system considerably dominates the drought-affected regions resulted in this study, especially for soybean (99% of the total calculated harvested area), maize (89%), and wheat (83%), while in the case of rice, the proportion of irrigated cropland is relatively higher (63%). Irrigation tends to mitigate rice yield loss as the centroid of the bins tends to be positive (yield gain), while rainfed rice tends to have yield loss. For the major rainfed crops (maize, soybean, wheat), there seems to be no considerable difference between irrigated and rainfed system responses; both exhibit mainly yield loss despite a large difference in cropland share. Nevertheless, the vast rainfed system is negatively impacted by drought, shown by an extensive area proportion attributable to yield loss, which indicates the substantial drought impact on this cropping system globally. Particularly, maize, soybean, and wheat are damaged by drought, equal to 65%, 69%, and 62% of the calculated harvested area. Therefore, we may argue that variability of irrigation fraction may not be adequate to explain crop yield sensitivity to drought owing to the dominance of a rainfed system over global croplands calculated in this study.

Further study may consider other important parameters for drought parameterization, such as temperature or evapotranspiration (ET), as they play a significant role in exacerbating drought impact through increasing evaporative demand (53,76). The drought indicator can also be represented by other drought parameters, such as growing season soil moisture conditions based on physical modeling or remote sensing estimates on a global scale (30,77). Previous studies reported that the impact of drought on agricultural production due to lacking soil moisture is often associated with extreme heat, posing a more severe risk to crops (53,78,79). Joint drought and heat events indicators may provide insight into these relationships (73,80,81). We suggest considering these possible mechanisms accounting for hydrological features on drought estimates (rather than only meteorological factors, e.g., precipitation), especially in some regions agricultural systems impose more complex atmospheric soil interaction and are heavily reliant on the other common water sources such as irrigation and groundwater (53,82).

Moreover, we indicate that the overall effect of socio-economic factors, i.e., GDP per capita, is relatively weak in determining crop-drought sensitivity—which may be underestimated in this study.

Specifically for soybean, higher GDP may slightly induce higher yield loss sensitivity to drought, while maize slightly responds differently. Previous studies (17,83) reported that higher GDP per capita generally reflects larger average farm sizes (84), which are typically market-oriented. Furthermore, a previous study (83) suggested that this type of system may be more sensitive to drought when they tend to achieve maximum yield potential associated with higher risk instead of adjusting low-risk management practices during climate stress. Possible reasons were highlighted: when GDP per capita increases, conventional drought coping strategies may be reduced, while in poorer regions, traditional and well-settled adaptation strategies may be more preserved, possibly reducing the harvested yield volatility (17,83). However, this socio-economic indicator effect in defining the crop sensitivity to drought may remain complex in actual conditions. In contrast, numerous pieces of literature have indicated that the importance of GDP or capital indicators may decrease vulnerability (i.e., increasing adaptive capacity) due to more financial reserves for investing in, e.g., drought-tolerant breeding, irrigation facility, mechanization, or weather forecasting (11,17,21,22). Other studies also highlighted that crop resilience to drought is higher in rich and developing countries than in middle-income countries (17). It is worth noting that this present study only considers a specific vulnerability measure (i.e., crop yield sensitivity, yield loss) based on the relationship between crop yield and meteorological drought. In contrast, a vulnerability in its comprehensive definition may consider more factors (i.e., drought-induced production and economic loss, food trade) (5,7).

Finally, we acknowledge the limitation of this study related to the datasets used, particularly the reliance on the gridded dataset of historical yield (GDHY) and SPI-based drought indicator as the two main input parameters. The crop-drought sensitivity patterns are also subject to change if other datasets (i.e., sub-country crop yield data (3)) and other drought indicators are used (30). The drought definition used in this study can be further improved by considering other parameters (i.e., temperature, ET, soil moisture). Therefore, we acknowledge that crop yield sensitivity here is exclusively determined by the specific datasets used here, mainly obtained from the SPI-based drought indicator and yield dataset from GDHY (34). It is noteworthy that the result of key determinants of the global pattern of crop sensitivity to drought may be sensitive to the data and model selection in this study.

At this point, challenges and questions remain for empirically understanding the complete drought characterization on global agriculture. Nevertheless, this study is worth undertaking since this provides the first attempt to broadly link several possible determinants with the crop-drought relationship to reveal the most influential parameter in defining the relationship patterns over the global scale. Overall, this study’s results may provide a basis for measures to achieve food security against the pressures such as increasing severity and frequency of extreme events under climate changes, food demand due to the growing global population, and land availability (85).

**Fig 4. Crop-drought sensitivity in each rainfed and irrigated defined by >80% of irrigated harvested area for maize, rice, soybean, and wheat.** The x-axis represents the bins of crop yield sensitivity, and the y-axis represents the percentage of the harvested area where data is available. The dashed line indicates the center value (zero), separating yield loss (negative) and yield gain (positive).

# 6. Conclusion

This paper explores key factors determining crop-drought sensitivity for maize, rice, soybean, and wheat from 1981 to 2016. Results suggest that drought impact on crops is notable globally and regionally along major global breadbaskets. We link the crop-drought sensitivity with various determinants using the Random Forest model. Results reveal that environmental factors (i.e., annual precipitation, soil water-holding capacity, soil acidity, annual potential evapotranspiration) and management factors (i.e., fertilizers application rate, growing season) are among the key factors in controlling crop sensitivity to drought. Our results may shed light on understanding global crop-drought sensitivity patterns and their key determinants. Future studies are expected to address the remaining gaps demonstrated in this study to understand crop system vulnerability to drought towards future challenges to achieve food security.

# References

1. Lobell DB, Field CB. Global scale climate-crop yield relationships and the impacts of recent warming. Environ Res Lett. 2007;2(1).
2. Vogel E, Donat MG, Alexander L V., Meinshausen M, Ray DK, Karoly D, et al. The effects of climate extremes on global agricultural yields. Environ Res Lett. 2019;14(5).
3. Ray DK, West PC, Clark M, Gerber JS, Prishchepov A V., Chatterjee S. Climate change has likely already affected global food production. PLoS One. 2019;14(5):1–18.
4. Iizumi T, Sakuma H, Yokozawa M, Luo JJ, Challinor AJ, Brown ME, et al. Prediction of seasonal climate-induced variations in global food production. Nat Clim Chang [Internet]. 2013;3(10):904–8. Available from: http://dx.doi.org/10.1038/nclimate1945
5. Kim W, Iizumi T, Nishimori M. Global patterns of crop production losses associated with droughts from 1983 to 2009. J Appl Meteorol Climatol. 2019;58(6):1233–44.
6. Hendrawan VSA, Kim W, Touge Y, Ke S, Komori D. A global-scale relationship between crop yield anomaly and multiscalar drought index based on multiple precipitation data. Environ Res Lett. 2022;17(1):014037.
7. IPCC. Climate Change 2007: Impacts, Adaptation and Vulnerability. Working Group II Contribution to the Intergovernmental Panel on Climate Change Fourth Assessment Report. Brussels, Belgium: IPCC; 2007.
8. Hasegawa T, Sakurai G, Fujimori S, Takahashi K, Hijioka Y, Masui T. Extreme climate events increase risk of global food insecurity and adaptation needs. Nat Food [Internet]. 2021;2(8):587–95. Available from: http://dx.doi.org/10.1038/s43016-021-00335-4
9. Liu D, Mishra AK, Ray DK. Sensitivity of global major crop yields to climate variables: A nonparametric elasticity analysis. Sci Total Environ [Internet]. 2020;748:141431. Available from: https://doi.org/10.1016/j.scitotenv.2020.141431
10. Simelton E, Fraser EDG, Termansen M, Forster PM, Dougill AJ. Typologies of crop-drought vulnerability: an empirical analysis of the socio-economic factors that influence the sensitivity and resilience to drought of three major food crops in China (1961-2001). Environ Sci Policy [Internet].

2009;12(4):438–52. Available from: http://dx.doi.org/10.1016/j.envsci.2008.11.005

11. Hagenlocher M, Meza I, Anderson CC, Min A, Renaud FG, Walz Y, et al. Drought vulnerability and risk assessments: State of the art, persistent gaps, and research agenda. Environ Res Lett. 2019;14(8).
12. Wilhelmi O V, Wilhite DA. Assessing Vulnerability to Agricultural Drought: A Nebraska Case Study. Nat Hazards [Internet]. 2002;25(1):37–58. Available from: https://doi.org/10.1023/A:1013388814894
13. Fahad S, Bajwa AA, Nazir U, Anjum SA, Farooq A, Zohaib A, et al. Crop production under drought and heat stress: Plant responses and management options. Front Plant Sci. 2017;8(June):1–16.
14. Rosenzweig C, Elliott J, Deryng D, Ruane AC, Müller C, Arneth A, et al. Assessing agricultural risks of climate change in the 21st century in a global gridded crop model intercomparison. Proc Natl Acad Sci U S A. 2014;111(9):3268–73.
15. Daryanto S, Wang L, Jacinthe PA. Global synthesis of drought effects on maize and wheat production. PLoS One. 2016;11(5):1–15.
16. Fraser EDG, Simelton E, Termansen M, Gosling SN, South A. “ Vulnerability hotspots” : Integrating socio-economic and hydrological models to identify where cereal production may decline in the future due to climate change induced drought. Agric For Meteorol [Internet]. 2013;170:195–205. Available from: http://dx.doi.org/10.1016/j.agrformet.2012.04.008
17. Simelton E, Fraser EDG, Termansen M, Benton TG, Gosling SN, South A, et al. The socioeconomics of food crop production and climate change vulnerability: a global scale quantitative analysis of how grain crops are sensitive to drought. Food Secur. 2012;4(2):163–79.
18. Antwi-Agyei P, Fraser EDG, Dougill AJ, Stringer LC, Simelton E. Mapping the vulnerability of crop production to drought in Ghana using rainfall, yield and socioeconomic data. Appl Geogr [Internet].

2012;32(2):324–34. Available from: http://dx.doi.org/10.1016/j.apgeog.2011.06.010

19. Lobell DB, Deines JM, Tommaso S Di. Changes in the drought sensitivity of US maize yields. Nat Food

[Internet]. 2020;1(11):729–35. Available from: http://dx.doi.org/10.1038/s43016-020-00165-w

20. Kamali B, Abbaspour KC, Wehrli B, Yang H. Drought vulnerability assessment of maize in Sub-Saharan

Africa: Insights from physical and social perspectives. Glob Planet Change [Internet]. 2018;162(June

2017):266–74. Available from: https://doi.org/10.1016/j.gloplacha.2018.01.011

21. Murthy CS, Laxman B, Sesha Sai MVR. Geospatial analysis of agricultural drought vulnerability using a composite index based on exposure, sensitivity and adaptive capacity. Int J Disaster Risk Reduct

[Internet]. 2015;12:163–71. Available from: http://dx.doi.org/10.1016/j.ijdrr.2015.01.004

22. Carrão H, Naumann G, Barbosa P. Mapping global patterns of drought risk: An empirical framework based on sub-national estimates of hazard, exposure and vulnerability. Glob Environ Chang. 2016;39:108–24.
23. Meza I, Siebert S, Döll P, Kusche J, Herbert C, Rezaei EE, et al. Global-scale drought risk assessment for agricultural systems. Nat Hazards Earth Syst Sci. 2020;20(2):695–712.
24. Williams PA, Crespo O, Abu M, Simpson NP. A systematic review of how vulnerability of smallholder agricultural systems to changing climate is assessed in Africa. Environ Res Lett. 2018;13(10).
25. McKee T, Doesken N, Kleist J. The relationship of drought frequency and duration to time scales. In: Proc 8th Conf on Applied Climatology. 1993. p. 179–83.
26. Wilhite DA, Glantz MH. Understanding: the Drought Phenomenon: The Role of Definitions. Water Int [Internet]. 1985 Jan 22;10(3):111–20. Available from:

https://www.taylorfrancis.com/books/9781000232257/chapters/10.4324/9780429301735-2

27. Turco M, Jerez S, Donat MG, Toreti A, Vicente-Serrano SM, Doblas-Reyes FJ. A global probabilistic dataset for monitoring meteorological droughts. Bull Am Meteorol Soc. 2020;101(10):E1628–44.
28. Mishra AK, Singh VP. A review of drought concepts. J Hydrol [Internet]. 2010;391(1–2):202–16. Available from: http://dx.doi.org/10.1016/j.jhydrol.2010.07.012
29. Spinoni J, Naumann G, Carrao H, Barbosa P, Vogt J. World drought frequency, duration, and severity for

1951–2010. Int J Clim [Internet]. 2014;34:2792–804. Available from: https://doi.org/10.1002/joc.3875

30. Hoffmann D, Gallant AJE, Arblaster JM. Uncertainties in Drought From Index and Data Selection. J Geophys Res Atmos. 2020;125(18):1–21.
31. Zargar A, Sadiq R, Naser B, Khan FI. A review of drought indices. Environ Rev. 2011;19(1):333–49.
32. Lloyd-Hughes B, Saunders MA. A drought climatology for Europe. Int J Climatol. 2002;22(13):1571–92.
33. Sacks WJ, Deryng D, Foley JA, Ramankutty N. Crop planting dates: An analysis of global patterns. Glob Ecol Biogeogr. 2010;19(5):607–20.
34. Iizumi T, Sakai T. The global dataset of historical yields for major crops 1981–2016. Sci Data [Internet].

2020;7(1):1–7. Available from: http://dx.doi.org/10.1038/s41597-020-0433-7

35. Lu J, Carbone GJ, Gao P. Detrending crop yield data for spatial visualization of drought impacts in the United States, 1895–2014. Agric For Meteorol [Internet]. 2017;237–238:196–208. Available from: http://dx.doi.org/10.1016/j.agrformet.2017.02.001
36. Liu H, Jin Y, Roche LM, O’Geen AT, Dahlgren RA. Understanding spatial variability of forage production in California grasslands: Delineating climate, topography and soil controls. Environ Res Lett. 2021;16(1).
37. Lychuk TE, Moulin AP, Lemke RL, Gossen BD, Leeson JY, Kirk A, et al. Effects of crop inputs, diversity, environment, and terrain on yield in an 18-yr study in the semi-arid Canadian prairies. Can J Plant Sci. 2017;97(4):715–30.
38. Davis KF, Chhatre A, Rao ND, Singh D, Defries R. Sensitivity of grain yields to historical climate variability in India. Environ Res Lett. 2019;14(6).
39. Iizumi T, Hosokawa N, Wagai R. Soil carbon-food synergy: sizable contributions of small-scale farmers. CABI Agric Biosci [Internet]. 2021;2(1):1–15. Available from: https://doi.org/10.1186/s43170-02100063-6
40. Williams A, Hunter MC, Kammerer M, Kane DA, Jordan NR, Mortensen DA, et al. Soil water holding capacity mitigates downside risk and volatility in US rainfed maize: Time to invest in soil organic matter? PLoS One. 2016;11(8):1–11.
41. Yamoah CF, Walters DT, Shapiro CA, Francis CA, Hayes MJ. Standardized precipitation index and nitrogen rate effects on crop yields and risk distribution in maize. Agric Ecosyst Environ. 2000;80(1– 2):113–20.
42. Portmann FT, Siebert S, Döll P. MIRCA2000-Global monthly irrigated and rainfed crop areas around the year 2000: A new high-resolution data set for agricultural and hydrological modeling. Global Biogeochem Cycles. 2010;24(1):n/a-n/a.
43. Siebert S, Döll P, Hoogeveen J, Faures J-M, Frenken K, Feick S. Development and validation of the global map of irrigation areas. Hydrol Earth Syst Sci [Internet]. 2005;9(5):535–47. Available from: https://hess.copernicus.org/articles/9/535/2005/
44. Harris I, Osborn TJ, Jones P, Lister D. Version 4 of the CRU TS monthly high-resolution gridded multivariate climate dataset. Sci Data. 2020;7(1):1–18.
45. IIASA/FAO. Global Agro-ecological Zones (GAEZ v3.0). Laxenburg, Austria and Rome, Italy.: IIASA and FAO; 2010.
46. Montzka C, Herbst M, Weihermüller L, Verhoef A, Vereecken H. A global data set of soil hydraulic properties and sub-grid variability of soil water retention and hydraulic conductivity curves. Earth Syst Sci Data. 2017;9(2):529–43.
47. Wieder W. Regridded Harmonized World Soil Database v1.2 [Internet]. ORNL Distributed Active Archive Center; 2014. Available from: http://daac.ornl.gov/cgi-bin/dsviewer.pl?ds_id=1247
48. Webb RS, Rosenzweig CE, Levine ER. Specifying land surface characteristics in general circulation models: Soil profile data set and derived water-holding capacities. Global Biogeochem Cycles. 1993;7(1):97–108.
49. Siebert S, Burke J, Faures JM, Frenken K, Hoogeveen J, Döll P, et al. Groundwater use for irrigation – a global inventory. Hydrol Earth Syst Sci [Internet]. 2010;14(10):1863–80. Available from:

https://hess.copernicus.org/articles/14/1863/2010/

50. Potter P, Ramankutty N, Bennett EM, Donner SD. Characterizing the Spatial Patterns of Global Fertilizer Application and Manure Production. Earth Interact [Internet]. 2010;14:1–22. Available from:

https://doi.org/10.1175/2009EI288.1

51. Kummu M, Taka M, Guillaume JHA. Gridded global datasets for Gross Domestic Product and Human Development Index over 1990-2015. Sci Data. 2018;5:1–15.
52. CIESIN. Gridded Population of the World, Version 4 (GPWv4): Population Density, Revision 11 [Internet]. Palisades, NY: NASA Socioeconomic Data and Applications Center (SEDAC); 2018. Available from: https://doi.org/10.7927/H49C6VHW
53. Lesk C, Coffel E, Winter J, Ray D, Zscheischler J, Seneviratne SI, et al. Stronger temperature–moisture couplings exacerbate the impact of climate warming on global crop yields. Nat Food [Internet].

2021;2(9):683–91. Available from: http://dx.doi.org/10.1038/s43016-021-00341-6

54. Breiman L. Random Forests. Mach Learn [Internet]. 2001;45(1):5–32. Available from:

https://doi.org/10.1023/A:1010933404324

55. Liaw A, Wiener M. Classification and Regression by randomForest. R News. 2002;2(3):18–22.
56. Friedman JH. Greedy function approximation: A gradient boosting machine. Ann Stat [Internet].

2001;29(5):1189–232. Available from: https://doi.org/10.1214/aos/1013203451

57. Nielsen DC, Vigil MF, Benjamin JG. The variable response of dryland corn yield to soil water content at planting. Agric Water Manag. 2009;96(2):330–6.
58. Vicente-Serrano SM. Evaluating the impact of drought using remote sensing in a Mediterranean, Semiarid Region. Nat Hazards. 2007;40(1):173–208.
59. Ray DK, Gerber JS, Macdonald GK, West PC. Climate variation explains a third of global crop yield variability. Nat Commun. 2015;6:1–9.
60. Strauss F, Moltchanova E, Schmid E. Spatially Explicit Modeling of Long-Term Drought Impacts on Crop Production in Austria. Am J Clim Chang. 2013;02(03):1–11.
61. Lambert L, Chitrakar BD. Variation of potential evapotranspiration with elevation in Nepal. Mt Res Dev. 1989;9(2):145–52.
62. He D, Wang E. On the relation between soil water holding capacity and dryland crop productivity. Geoderma. 2019;353(January):11–24.
63. Sweet SK, Wolfe DW, DeGaetano A, Benner R. Anatomy of the 2016 drought in the Northeastern United States: Implications for agriculture and water resources in humid climates. Agric For Meteorol [Internet].

2017;247(June):571–81. Available from: http://dx.doi.org/10.1016/j.agrformet.2017.08.024

64. Wang J, Wang X, Wang J. Profile distribution of CO2 in an arid saline-alkali soil with gypsum and wheat straw amendments: a two-year incubation experiment. Sci Rep [Internet]. 2018;8(1):1–10. Available from: http://dx.doi.org/10.1038/s41598-018-30312-0
65. Shao Y, Irannejad P. On the choice of soil hydraulic models in land-surface schemes. Boundary-Layer Meteorol. 1999;90(1):83–115.
66. Solone R, Bittelli M, Tomei F, Morari F. Errors in water retention curves determined with pressure plates: Effects on the soil water balance. J Hydrol [Internet]. 2012;470–471:65–74. Available from: http://dx.doi.org/10.1016/j.jhydrol.2012.08.017
67. Bodner G, Nakhforoosh A, Kaul HP. Management of crop water under drought: a review. Agron Sustain Dev. 2015;35(2):401–42.
68. Samar Raza MA, Farrukh Saleem M, Mustafa Shah G, Jamil M, Haider Khan I. Potassium applied under drought improves physiological and nutrient uptake performances of wheat (Triticum aestivun L.). J Soil Sci Plant Nutr. 2013;13(1):175–85.
69. Ul-Allah S, Ijaz M, Nawaz A, Sattar A, Sher A, Naeem M, et al. Potassium Application Improves Grain Yield and Maize Hybrids. Plants. 2020;9(75):1–11.
70. Jin J, Wang G, Liu X, Pan X, Herbert SJ, Tang C. Interaction Between Phosphorus Nutrition and Drought on Grain Yield, and Assimilation of Phosphorus and Nitrogen in Two Soybean Cultivars Differing in Protein Concentration in Grains. J Plant Nutr [Internet]. 2006;29(8):1433–49. Available from: https://doi.org/10.1080/01904160600837089
71. Wang M, Zheng Q, Shen Q, Guo S. The critical role of potassium in plant stress response. Int J Mol Sci. 2013;14(4):7370–90.
72. Lu J, Carbone GJ, Huang X, Lackstrom K, Gao P. Mapping the sensitivity of agriculture to drought and estimating the effect of irrigation in the United States, 1950–2016. Agric For Meteorol [Internet].

2020;292–293(March):108124. Available from: https://doi.org/10.1016/j.agrformet.2020.108124

73. Matiu M, Ankerst DP, Menzel A. Interactions between temperature and drought in global and regional crop yield variability during 1961-2014. PLoS One. 2017;12(5):1–23.
74. Lesk C, Rowhani P, Ramankutty N. Influence of extreme weather disasters on global crop production. Nature [Internet]. 2016;529(7584):84–7. Available from: http://dx.doi.org/10.1038/nature16467
75. Troy TJ, Kipgen C, Pal I. The impact of climate extremes and irrigation on US crop yields. Environ Res Lett. 2015;10(5).
76. Rigden AJ, Mueller ND, Holbrook NM, Pillai N, Huybers P. Combined influence of soil moisture and atmospheric evaporative demand is important for accurately predicting US maize yields. Nat Food

[Internet]. 2020;1(2):127–33. Available from: http://dx.doi.org/10.1038/s43016-020-0028-7

77. Carrão H, Russo S, Sepulcre-Canto G, Barbosa P. An empirical standardized soil moisture index for agricultural drought assessment from remotely sensed data. Int J Appl Earth Obs Geoinf [Internet].

2016;48:74–84. Available from: http://dx.doi.org/10.1016/j.jag.2015.06.011

78. Berg A, Lintner BR, Findell K, Seneviratne SI, Denhurk B Van, Ducharne A, et al. Interannual coupling between summertime surface temperature and precipitation over land: Processes and implications for climate change. J Clim. 2015;28(3):1308–28.
79. Seneviratne SI, Corti T, Davin EL, Hirschi M, Jaeger EB, Lehner I, et al. Investigating soil moistureclimate interactions in a changing climate: A review. Earth-Science Rev [Internet]. 2010;99(3–4):125–61. Available from: http://dx.doi.org/10.1016/j.earscirev.2010.02.004
80. Lesk C, Anderson W. Decadal variability modulates trends in concurrent heat and drought over global croplands. Environ Res Lett [Internet]. 2021 May 1;16(5):055024. Available from:

https://iopscience.iop.org/article/10.1088/1748-9326/abeb35

81. Lipiec J, Doussan C, Nosalewicz A, Kondracka K. Effect of drought and heat stresses on plant growth and yield: A review. Int Agrophysics. 2013;27(4):463–77.
82. Zipper SC, Qiu J, Kucharik CJ. Drought effects on US maize and soybean production: Spatiotemporal patterns and historical changes. Environ Res Lett. 2016;11(9).
83. Stringer LC, Fraser EDG, Harris D, Lyon C, Pereira L, Ward CFM, et al. Adaptation and development pathways for different types of farmers. Environ Sci Policy [Internet]. 2020;104(April 2019):174–89. Available from: https://doi.org/10.1016/j.envsci.2019.10.007
84. Lowder SK, Skoet J, Raney T. The Number, Size, and Distribution of Farms, Smallholder Farms, and Family Farms Worldwide. World Dev [Internet]. 2016;87:16–29. Available from:

http://dx.doi.org/10.1016/j.worlddev.2015.10.041

85. United Nations. Transforming our world : the 2030 Agenda for Sustainable Development [Internet]. Vol. 16301. 2015 [cited 2022 Apr 7]. p. 1–35. Available from:

https://www.refworld.org/docid/57b6e3e44.html

# Supporting information

**S1 Fig. Example of the linear regression between yield anomaly (**𝚫𝒀**) and** 𝑫𝑰 **in a grid cell.** Cropdrought sensitivity (*β*) is obtained based on the slope coefficient.

**S2 Fig. Crop yield sensitivity to drought based on 9-month SPI across global croplands based on**

**GDHY for maize, rice, soybean, and wheat.** Sensitivity is calculated by the linear slope coefficient (*β*) with crop yield anomaly (%) per drought magnitude. White grid cells show no cropland or crop yield data, and grey shows the non-significant grid cells (𝑃 ≥ 0.05).

**S3 Fig. Relationship between crop-drought sensitivity (*β*) as the response variable and the determinants factors as independent variables based on each partial dependence using Random Forest models.** The order shows high to lower average relative importance across crops based on this specific model (not overall). The lower negative y-axis indicates higher yield loss due to drought.

[Click here to access/download;Figure;Fig1.ti](https://www.editorialmanager.com/pone/download.aspx?id=31772740&guid=a7f88418-8587-4c53-86ef-c3357f42b372&scheme=1)

[Click here to access/download;Figure;Fig2.tif](https://www.editorialmanager.com/pone/download.aspx?id=31772739&guid=e1c59bf1-2b50-44cd-8f45-dc1e849d178f&scheme=1)

[Click here to access/download;Figure;Fig3.tif](https://www.editorialmanager.com/pone/download.aspx?id=31772729&guid=c9b160a5-527f-4bab-9a80-36a61f12b4fb&scheme=1)

[Click here to access/download;Figure;Fig4.ti](https://www.editorialmanager.com/pone/download.aspx?id=31772727&guid=cb5f98d0-8aba-4bc9-9c11-d7fd5ae57de9&scheme=1)

[Click here to access/download](https://www.editorialmanager.com/pone/download.aspx?id=31772933&guid=37074a71-2406-4b99-abea-49ea131c9513&scheme=1)

[Supporting Information S1_Fig.tif](https://www.editorialmanager.com/pone/download.aspx?id=31772933&guid=37074a71-2406-4b99-abea-49ea131c9513&scheme=1)

[Click here to access/download](https://www.editorialmanager.com/pone/download.aspx?id=31772932&guid=f87f0e67-0123-419a-a498-1e50be0834fb&scheme=1)

[Supporting Information S2_Fig.tif](https://www.editorialmanager.com/pone/download.aspx?id=31772932&guid=f87f0e67-0123-419a-a498-1e50be0834fb&scheme=1)

[Click here to access/download](https://www.editorialmanager.com/pone/download.aspx?id=31772926&guid=14ca2720-e079-4a13-952e-617c2e4132fb&scheme=1)

[Supporting Information S3_Fig.tif](https://www.editorialmanager.com/pone/download.aspx?id=31772926&guid=14ca2720-e079-4a13-952e-617c2e4132fb&scheme=1)
